# Correcting for selection bias in HIV prevalence estimates: an application of sample selection models using data from population‐based HIV surveys in seven sub‐Saharan African countries

**DOI:** 10.1002/jia2.25954

**Published:** 2022-08-05

**Authors:** Anton M. Palma, Giampiero Marra, Rachel Bray, Suzue Saito, Anna Colletar Awor, Mohamed F. Jalloh, Alexander Kailembo, Wilford Kirungi, George S. Mgomella, Prosper Njau, Andrew C. Voetsch, Jennifer A. Ward, Till Bärnighausen, Guy Harling

**Affiliations:** ^1^ ICAP at Columbia University New York New York USA; ^2^ Institute for Clinical and Translational Sciences University of California Irvine Irvine California USA; ^3^ Department of Statistics University College London London UK; ^4^ Division of Global HIV and Tuberculosis Center for Global Health CDC Kampala Uganda; ^5^ Division of Global HIV and Tuberculosis Center for Global Health CDC Dar es Salaam Tanzania; ^6^ National Institutes of Health National Institute of Dental and Craniofacial Research Bethesda Maryland USA; ^7^ Uganda Ministry of Health Kampala Uganda; ^8^ University of Cambridge Cambridge UK; ^9^ U.S. Centers for Disease Control and Prevention Atlanta Georgia USA; ^10^ Africa Health Research Institute KwaZulu‐Natal South Africa; ^11^ MRC/Wits Rural Public Health & Health Transitions Research Unit (Agincourt) University of the Witwatersrand Johannesburg South Africa; ^12^ Department of Epidemiology & Harvard Center for Population and Development Studies Harvard T.H. Chan School of Public Health Boston Massachusetts USA; ^13^ Heidelberg Institute of Global Health University of Heidelberg Heidelberg Germany; ^14^ Institute for Global Health University College London London UK

**Keywords:** HIV prevalence, population surveys, missing data, selection models, non‐participation, surveillance

## Abstract

**Introduction:**

Population‐based biomarker surveys are the gold standard for estimating HIV prevalence but are susceptible to substantial non‐participation (up to 30%). Analytical missing data methods, including inverse‐probability weighting (IPW) and multiple imputation (MI), are biased when data are missing‐not‐at‐random, for example when people living with HIV more frequently decline participation. Heckman‐type selection models can, under certain assumptions, recover unbiased prevalence estimates in such scenarios.

**Methods:**

We pooled data from 142,706 participants aged 15–49 years from nationally representative cross‐sectional Population‐based HIV Impact Assessments in seven countries in sub‐Saharan Africa, conducted between 2015 and 2018 in Tanzania, Uganda, Malawi, Zambia, Zimbabwe, Lesotho and Eswatini. We compared sex‐stratified HIV prevalence estimates from unadjusted, IPW, MI and selection models, controlling for household and individual‐level predictors of non‐participation, and assessed the sensitivity of selection models to the copula function specifying the correlation between study participation and HIV status.

**Results:**

In total, 84.1% of participants provided a blood sample to determine HIV serostatus (range: 76% in Malawi to 95% in Uganda). HIV prevalence estimates from selection models diverged from IPW and MI models by up to 5% in Lesotho, without substantial precision loss. In Tanzania, the IPW model yielded lower HIV prevalence estimates among males than the best‐fitting copula selection model (3.8% vs. 7.9%).

**Conclusions:**

We demonstrate how HIV prevalence estimates from selection models can differ from those obtained under missing‐at‐random assumptions. Further benefits include exploration of plausible relationships between participation and outcome. While selection models require additional assumptions and careful specification, they are an important tool for triangulating prevalence estimates in surveys with substantial missing data due to non‐participation.

## 1 INTRODUCTION

Accurate HIV prevalence estimates are crucial for monitoring HIV disease burden and guiding testing and clinical management. However, population‐based HIV biomarker surveys, the gold standard for estimating HIV prevalence, can be prone to substantial non‐participation that is non‐random [1]. Past biomarker non‐response rates have been as high as 30% in nationally representative Demographic and Health Surveys (DHS) and other population‐based studies [2, 3, 4]. When participants and non‐participants differ with respect to HIV status, naïve use of prevalence data from surveys yields biased results [1]. HIV‐seropositive persons may decline to test more often for various reasons. Individuals who know their serostatus may want to avoid unnecessary re‐testing [5]; those who do not know, but suspect their status, may decline to test for fear of disclosure and HIV‐related stigma or to prevent imposing burdens on family or caregivers [6].

Growing attention has turned to analytical methods to deal with non‐ignorable missing data, such as those that are missing not at random (MNAR). If data are missing completely at random, no adjustment is needed. When HIV status is missing at random (MAR) conditional on observed variables, the use of inverse‐probability weighting (IPW) or multiple imputation (MI) is sufficient to generate unbiased estimates [7, 8]. However, it is not possible to empirically verify whether data are indeed MAR or instead MNAR, where unwillingness to consent to biomarker testing is associated with HIV status (actual or perceived) due to unmeasured factors. In such scenarios, HIV prevalence estimates are susceptible to bias even when using IPW or MI methods.

One method that can potentially recover unbiased prevalence estimates from MNAR data is the Heckman‐type sample selection model [9, 10]. Selection models operate by using the association between the probability of selection (HIV test participation) and the outcome of interest (HIV serostatus) and jointly estimating population‐level prevalence for participants and non‐participants. Selection models empirically require one or more selection variables—that is, variables that predict the outcome only via their association with the participation process. Previous work has proposed that interviewer identity fits this criterion for biomarker‐measured outcomes, since interviewer skill and personality may affect a participant's willingness to consent for testing but could not plausibly affect their HIV status; moreover, interviewer identity is commonly available in survey data [11].

Early selection models required bivariate normally distributed errors for selection and outcome, but this requirement has now been relaxed by specifying a copula function that describes the dependence between the error terms in the outcome and selection models [12]. This development has enhanced the flexibility of selection models and may even increase their utility as a means to identify the form of non‐participation biases in a given population, yielding insights for HIV prevention [13]. Such methodological advancements provide support for the routine adoption of selection models in epidemiological research. However, despite extensive use in economics, selection models have been used sparingly in epidemiology and typically using earlier formulations with less flexible modelling assumptions [11, 14, 15, 16, 17].

Selection models are particularly relevant for Population‐based HIV Impact Assessments (PHIAs); these are nationally representative cross‐sectional household‐based surveys aimed to capture the state of the HIV epidemic in the most‐affected countries, such as sub‐Saharan African countries and Haiti [18]. PHIA surveys include HIV testing via venous blood draw and other biomarker testing. PHIA response rates were generally higher than other comparable nationally representative surveys [2]. Nonetheless, blood test refusal rates in some countries have been as high as 30% for men and 19% for women [19, 20, 21, 22, 23, 24, 25]. While published PHIA estimates are weighted to account for non‐response and the complex sampling design, these estimates relied on observed covariates and thus may not account for non‐response associated with HIV status or perceptions around HIV status.

In this study, we aimed to test whether HIV prevalence estimates in seven PHIA surveys conducted in Tanzania, Uganda, Malawi, Zambia, Zimbabwe, Lesotho and Eswatini were sensitive to the method chosen to handle missing data. We compared sex‐stratified HIV prevalence estimates in each country, using naïve, IPW, MI and sample selection models, to evaluate the potential benefit of accounting for MNAR data in such estimates.

## 2 METHODS

### 2.1 Data source and sampling

Each PHIA survey was designed to characterize HIV prevalence and incidence, as well as progress towards The Joint United Nations Programme on HIV/AIDS (UNAIDS) 90‐90‐90 targets, referring to 90% of people living with HIV knowing their HIV status, 90% of those who know their status receiving antiretroviral therapy (ART) and 90% of those on ART achieving viral suppression [26]. Similar to DHS, the sampling approach used in PHIA begins with a first‐stage sampling of census enumeration areas (EAs) with probability proportional to size within geographic strata defined by the highest sub‐national geographic designated area in each country, such as province or region. Within sampled EAs, households were randomly selected to participate in a interview to provide household information and a household membership roster. All rostered adults meeting country‐specific age criteria (typically between 15 and 64 years of age) who had slept in the household the night before the survey were eligible to participate in an individual interview. Consenting participants were offered venous blood sample collection to conduct HIV rapid testing. Consent for the interview was obtained prior to the interview, and consent for the blood test was obtained after the interview immediately prior to testing, except in Eswatini where both consents were elicited prior to the interview. HIV test results were returned to respondents at the time of the survey, and those testing seropositive and a small sample of those testing seronegative were further provided point‐of‐care CD4 testing, referral to HIV care and treatment, and viral load testing, the results of which were returned to nearby health facilities within 6–12 weeks [18, 19, 20, 21, 22, 23, 24, 25].

### 2.2 Measures

We defined HIV test participation as the completion of blood testing based on each country's national HIV testing algorithm; this involved sequential testing that utilized up to three HIV rapid tests administered at the home. HIV seropositivity was defined as blood test‐confirmed HIV‐positive status using a confirmatory HIV test; negative and indeterminate test results were classified as HIV seronegative. We selected potential covariates for inclusion in MI and selection models on the basis of hypothesized association with HIV test participation or HIV status from the existing literature. Selected covariates included: urban residential location (urban vs. rural); 5‐year age‐group (15‐19, 20–24, 25–29, 30–34, 35–39, 40–44 or 45–49); marital status (never married, married or living together, divorced or separated, or widowed); educational attainment (no education, primary, secondary, more than secondary); ethnicity (categorical, when available); household wealth quintile within the country (based on household assets and characteristics that varied across countries [18, 27]); circumcised versus uncircumcised (males only); pregnant versus not pregnant (females only); self‐reported ever versus never had sex; self‐reported ever versus never received HIV testing prior to the survey; province/region; and language.

Interviewers’ identities were recorded using unique anonymized IDs. Two different interviewers may have administered the interview and blood draw depending on training in phlebotomy and HIV testing. If the interview was administered by an interviewer without clinical training, consent for blood draw was obtained by a separate interviewer [18]. Among individuals who participated in the individual interview and were offered a blood test, we used the identity of the interviewer who elicited consent for blood testing. Among participants who did not participate in the interview, we used the identity of the interviewer who elicited consent for the interview.

### 2.3 Analysis

All rostered de facto household members in participating households aged 15–49 years were potentially eligible for analysis. Sample characteristics were described among the entire sample and compared to those who completed an individual interview and the further subset who completed a blood test. Blood test participation rates were calculated overall and by country, sex and age.

We estimated sex‐stratified HIV prevalence and 95% confidence intervals (CIs). All prevalence estimates were calculated using survey design weights to adjust for EA and household selection probabilities and household non‐participation. This weighting approach allowed at minimum for all rostered de facto household members to represent the national population. Additional adjustment for interview and blood test non‐participation was performed under models with different missing data approaches as described below. All analyses were conducted using the MICE and GJRM packages in R v3.6.2 [28, 29].

#### 2.3.1 Naïve model and maximal bounds

In naïve models, we calculated HIV prevalence as the weighted proportion of blood test participants who tested seropositive, with no further adjustment for individual‐level non‐participation. We also calculated maximal bounds, which represent lower and upper limits in the observed sample assuming all non‐participants were HIV seronegative or HIV seropositive, respectively [30].

#### 2.3.2 IPW model

IPW model estimates were additionally weighted to adjust for non‐participation in the individual interview and blood testing. Survey weights were constructed using the least absolute shrinkage and selection operation (LASSO) regression and chi‐squared automatic interaction detector (CHAID) tree classification algorithms to select a minimal set of available variables that best predict non‐response. The inverse probability of participation within strata defined by the identified set of variables was used to weight blood test respondents. This IPW approach was similar to the estimation method used in published PHIA reports [18, 31], except that we omitted post‐stratification weighting and used Taylor series variance estimation instead of jackknife repeated replication in order to simplify comparison between models.

#### 2.3.3 MI model

MI was performed using chained equations. Selected covariates were included in country‐ and sex‐specific imputation models to generate 20 imputation datasets each. HIV prevalence was estimated by running models across datasets and pooling results as per Rubin's rules [32]. Interview and blood test non‐participation weights were not used as individual‐level non‐response was accounted for by MI.

#### 2.3.4 Sample selection model

Selection models require specification of two equations, a selection equation for test participation and an outcome equation for HIV status [11, 12]. We used a probit regression model for binary HIV status $y_{i}$ for individual *i*:

$$y_{i}^{*}=X_{i}^{T}\beta +\varepsilon_{i}$$

$$\begin{array}{c} y_{i}=\left\{\begin{array}{c} 1\;\mathrm{if}\;y_{i}^{*}>0\\ 0\;\mathrm{otherwise} \end{array}\right. \end{array}$$

where $y_{i}^{*}$ is an unobserved latent variable determining the likelihood of having HIV‐positive status that depends on a vector of observed characteristics $X_{i}$ and random error $\varepsilon_{i}$ Similarly, we used a probit model for selection $s_{i}$ for individual *i*:

$$s_{i}^{*}=X_{i}^{T}\gamma +Z_{i}^{T}\phi +u_{i}$$

$$\begin{array}{c} s_{i}=\left\{\begin{array}{c} 1\;\mathrm{if}\;s_{i}^{*}>0\\ 0\;\mathrm{otherwise} \end{array}\right. \end{array}$$

where $s_{i}^{*}$ is an unobserved latent variable determining the likelihood of participation that depends on a vector of observed characteristics $x_{i}$, a potentially null vector of selection variables $Z_{i}$ and random error $u_{i}$. The predictors in $X_{i}$ were the same in both models, though this is not theoretically necessary. However, due to HIV‐related stigma, risk factors for HIV are likely to be predictors of non‐participation as well. Detailed information on the selected covariates in all models is provided in Table S1. Selection variables $Z_{i}$ are chosen on the basis of their predictive utility for participation but not the outcome, and are included in order to prevent collinearity between selection and outcome models [11]. Replicating prior studies, we used interviewer identity reflecting the fact that interviewer skill and personality may influence participation in HIV testing but could not plausibly affect HIV status [33], modelled as a random effect using a ridge penalty to improve convergence properties [12]. Province/region was modelled as a random effect using a Markov random field smoother [12].

We incorporated a copula function to model various forms of dependence between selection and outcome model errors [34]. We considered 19 different copulae: normal; Student's‐*t*; Frank; Ali–Miqhail–Haq; Pluckett; Farlie–Gumbel–Morgenstern; Hougaard; Joe; Gumbel; and Clayton—the last three each rotated by 0, 90, 180 and 270 degrees. We fit sample selection models using each copula function and selected the best‐fitting model using Akaike's information criterion (AIC). We explored how HIV prevalence estimates varied across copulae to assess the sensitivity of results to this model specification.

### 2.4 Sensitivity analyses

We conducted sensitivity analyses running all models only among individual interview participants. Compared to analyses among all eligible household members, these analyses were all additionally weighted for interview non‐response at minimum. Consequently, comparisons between models accounted only for differences in missingness assumptions with respect to blood test non‐participation.

### 2.5 Ethical approval

The PHIA protocol and data collection tools were approved by institutional review boards at Columbia University Medical Center, the United States Centers for Disease Control and Prevention and ethical review boards in each country.

## 3 RESULTS

### 3.1 Sample characteristics

**Figure 1:**
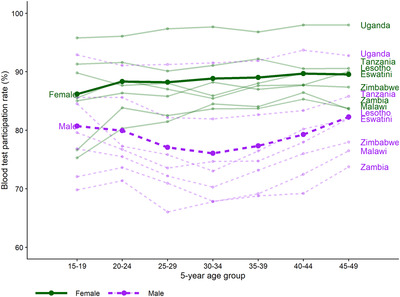
Blood test participation by country, sex and 5‐year age group. Each point indicates the blood test participation rate (% of all eligible adult household members) for each country by sex and 5‐year age group, and lines indicate the trends across age. Bolded points and lines indicate the pooled blood test participation rate for all countries by sex and 5‐year age group.

Across seven countries, *N* = 142,706 individuals aged 15–49 were eligible to participate (men: 64,049; women: 78,657). Overall, 90% (*N* = 129,033) participated in the individual interview (men: 54,900; women: 74,133); of these, 93% (*N* = 119,977) participated in blood testing, yielding an overall blood test participation rate of 84.1% of all de facto household members, (men: 79.0%; women: 88.2%). Participation rates differed by country, as well as sex and age, with lower participation among men and those 25–39 years old (Figure 1 and Table 1). Sample characteristics by country are reported in Tables S2a–2g.

**Table 1:** Pooled sample characteristics

	Men			Women		
	All eligible household members	Interview participants	Blood test participants	All eligible household members	Interview participants	Blood test participants
	*N* (% of total sample)	% of eligible	% of eligible	% of total sample	% of eligible	% of eligible
Total sample	64,049 (100%)	85.7% (54,900)	79.0% (50,584)	78,657 (100%)	94.2% (74,133)	88.2% (69,393)
Country						
Tanzania	13,059 (20.4%)	88.3%	84.0%	16,057 (20.4%)	94.9%	91.1%
Uganda	11,788 (18.4%)	93.5%	92.1%	15,200 (19.3%)	97.8%	96.8%
Malawi	9,030 (14.1%)	80.7%	69.8%	11,006 (14.0%)	92.7%	81.3%
Zambia	10,358 (16.2%)	80.2%	71.0%	12,192 (15.5%)	90.6%	82.1%
Zimbabwe	9,702 (15.1%)	82.7%	74.6%	11,806 (15.0%)	93.9%	86.6%
Lesotho	5,473 (8.5%)	86.8%	76.7%	6,871 (8.7%)	95.1%	87.2%
Eswatini	4,639 (7.2%)	86.0%	78.8%	5,525 (7.0%)	93.4%	88.3%
Urbanicity						
Urban^a^	22,684 (35.4%)	80.5%	72.1%	28,978 (36.8%)	93.3%	86.3%
Rural	41,365 (64.6%)	88.6%	82.7%	49,679 (63.2%)	94.8%	89.3%
Age group						
15–19	15,574 (24.3%)	85.8%	80.7%	16,654 (21.2%)	91.1%	86.2%
20–24	11,520 (18.0%)	87.0%	80.0%	15,619 (19.9%)	94.7%	88.3%
25–29	9,801 (15.3%)	84.9%	77.1%	13,222 (16.8%)	95.1%	88.2%
30–34	8,849 (13.8%)	83.8%	76.1%	11,423 (14.5%)	95.4%	88.8%
35–39	7499 (11.7%)	84.6%	77.4%	9,183 (11.7%)	95.5%	89.0%
40–44	6155 (9.6%)	86.3%	79.3%	7,203 (9.2%)	95.2%	89.7%
45–49	4651 (7.3%)	88.4%	82.3%	5,353 (6.8%)	95.1%	89.5%

Note: Numbers of men and women in the pooled sample, by country, urban/rural and 5‐year age groups. Percentages of total sample represent the proportion of the total number of eligible household members in the total pooled sample (column percent). Percent of eligible represents the row percent of eligible household members who participated in the interview and blood test, respectively. ^a^While most countries had two categories for urban and rural, in Lesotho, peri‐urban was treated as urban for analysis.

Consent rates varied widely by interviewer, ranging between 60% and 100%. Interviewer‐level consent rates were significantly positively associated with HIV prevalence in Eswatini, Zambia and Zimbabwe, but not in Malawi, Lesotho, Tanzania or Uganda (Table S3 and Figure S1).

### 3.2 HIV prevalence estimates

**Figure 2:**
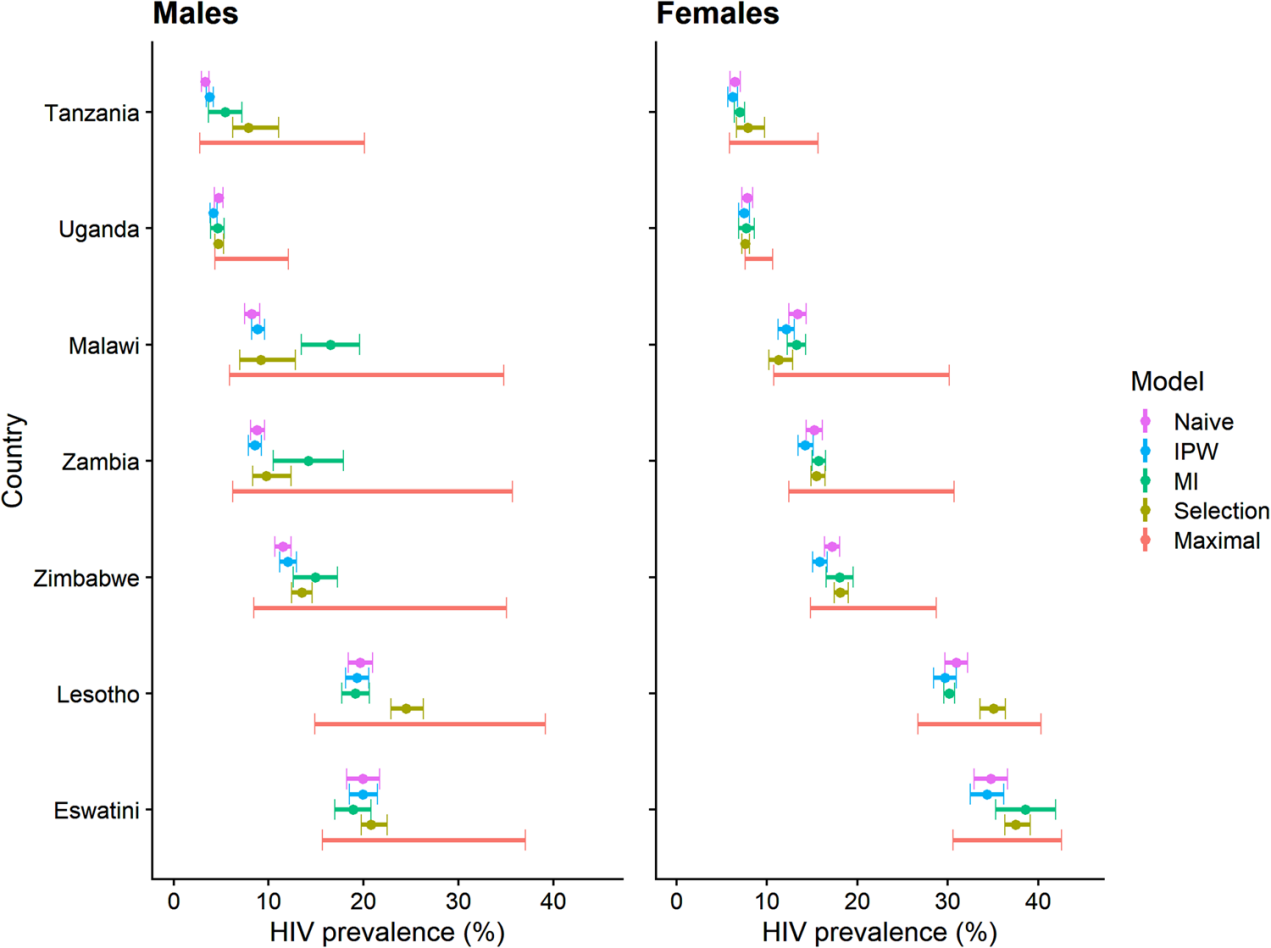
HIV prevalence estimates among adults aged 15–49 using different missing data methods. Abbreviations: IPW, inverse probability weighting; MI, multiple imputation. Points and error bars represent HIV prevalence (%) and 95% CIs obtained from models under various missing data treatments using data from all rostered household members. Maximal bounds are the theoretical minimum and maximum of possible HIV prevalence estimates in the sample assuming that all non‐participants were HIV negative or HIV positive. CIs from other models may exceed the maximal bounds due to Taylor series variance approximation.

Figure 2 and Table 2 show sex‐stratified national HIV prevalence estimates from each model. Naïve model estimates among men ranged from 3.3% in Tanzania to 19.9% in Eswatini and among women from 6.5% in Tanzania to 34.8% in Eswatini. IPW estimates were similar to those in the naïve models among men ranging from 3.8% in Tanzania to 19.9% in Eswatini and among women from 6.2% in Tanzania to 34.3% in Eswatini. Naïve and IPW point estimates did not differ by more than 1.3% in any survey. MI model estimates were substantially higher than IPW models among men in Tanzania (5.4% vs. 3.8%), Malawi (16.5% vs. 8.9%), Zambia (14.2% vs. 8.5%) and Zimbabwe (14.9% vs. 12.0%) and among women in Eswatini (38.6% vs. 34.3%).

**Table 2:** HIV prevalence estimates using different missingness assumptions, among all adults aged 15–49 who were eligible household members

	Country						
Model	Tanzania	Uganda	Malawi	Zambia	Zimbabwe	Lesotho	Eswatini
	Male						
	*N* = 10,971/13,059	*N* = 10,854/13,059	*N* = 6,306/9,030	*N* = 7358/10,358	*N* = 7,241/9,702	*N* = 4,199/5,473	*N* = 3,655/4,639
Naive	3.3 (2.9, 3.8)	4.7 (4.3, 5.2)	8.2 (7.5, 9.1)	8.8 (8.1, 9.6)	11.5 (10.6, 12.4)	19.7 (18.4, 21.0)	19.9 (18.2, 21.7)
IPW	3.8 (3.4, 4.2)	4.2 (3.8, 4.6)	8.9 (8.2, 9.6)	8.5 (7.9, 9.2)	12.0 (11.2, 12.9)	19.3 (18.1, 20.6)	19.9 (18.5, 21.5)
MI	5.4 (3.7, 7.2)	4.6 (3.9, 5.3)	16.5 (13.4, 19.6)	14.2 (10.5, 17.9)	14.9 (12.6, 17.3)	19.1 (17.7, 20.6)	18.9 (17.0, 20.8)
Selection	7.9 (6.0, 10.6)	4.7 (4.4, 5.3)	9.2 (7.1, 13.0)	9.7 (8.3, 12.5)	13.5 (12.3, 14.6)	24.3 (22.6, 26.6)	20.8 (19.9, 22.5)
Maximal	(2.7, 20.1)	(4.4, 12.1)	(5.9, 34.8)	(6.2, 35.7)	(8.4, 35.1)	(14.9, 39.1)	(15.7, 37.0)
	Female						
	*N* = 14,629/16,057	*N* = 14,716/15,200	*N* = 8,949/11,006	*N* = 10,010/12,192	*N* = 10,221/11,806	*N* = 5,990/6,871	*N* = 4,878/5,525
Naive	6.5 (6.0, 7.1)	7.8 (7.2, 8.5)	13.4 (12.5, 14.4)	15.2 (14.4, 16.1)	17.2 (16.4, 18.1)	30.9 (29.7, 32.2)	34.8 (32.9, 36.6)
IPW	6.2 (5.7, 6.8)	7.5 (6.9, 8.1)	12.1 (11.3, 13.1)	14.3 (13.4, 15.1)	15.9 (15.1, 16.7)	29.7 (28.5, 30.9)	34.3 (32.5, 36.2)
MI	7.0 (6.4, 7.6)	7.7 (6.9, 8.6)	13.3 (12.3, 14.3)	15.7 (15.0, 16.5)	18.1 (16.6, 19.6)	30.2 (29.6, 30.9)	38.6 (35.3, 41.9)
Selection	7.9 (6.7, 9.6)	7.6 (7.1, 8.1)	11.3 (10.2, 12.7)	15.5 (14.9, 16.4)	18.1 (17.6, 19.1)	34.9 (33.5, 37.0)	37.5 (36.3, 38.9)
Maximal	(5.9, 15.7)	(7.5, 10.7)	(10.8, 30.2)	(12.5, 30.7)	(14.8, 28.7)	(26.7, 40.3)	(30.6, 42.6)
	Female‐to‐male prevalence ratio						
Naive	1.97	1.66	1.63	1.73	1.50	1.57	1.75
IPW	1.63	1.79	1.36	1.68	1.33	1.54	1.72
MI	1.30	1.67	0.81	1.11	1.21	1.58	2.04
Selection	1.00	1.62	1.23	1.60	1.34	1.44	1.80

Note: N's indicate the number of blood test participants over the number of eligible persons. The eligible population includes all de facto household members at the top and all individuals who participated in the individual interview at the bottom.

Abbreviations: IPW, inverse probability weight; MI, multiple imputation.

**Figure 3:**
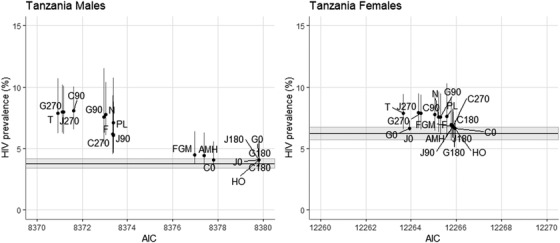
Comparison of HIV prevalence estimates from selection models under various copulae versus the IPW model. Points and error bars show selection model results by sex for each bivariate copula, AIC (x‐axis) versus HIV prevalence (%) and 95% CI (y‐axis). Horizontal black line and gray bar indicate the IPW estimate and 95% CI. Points further left exhibit better model fit. Point labels denote which copula was used: “N” = Normal, “F” = Frank, “T” = Student's‐*t*, “FGM” = Farlie–Gumbel–Morgenstern, “AMH” = Ali–Mikhail–Haq, “PL” = Placket, “HO” = Hougaard, “J” = Joe, “C” = Clayton, “G” = Gumbel. For Joe, Clayton and Gumbel copulae, numbers indicate rotation, either 0, 90, 180 or 270 degrees.

Figure 3 shows an example of selection model copula testing in Tanzania. For men, HIV prevalence estimates from selection models under all copulae were higher than the IPW model estimate of 3.8% but two groups of estimates emerged. One group yielded similar HIV prevalence estimates to the IPW model of around 4–5%, while the second group yielded HIV prevalence estimates between 6% and 8% with CIs that did not overlap that of the IPW estimate and had substantially better AIC‐based model fit. For women, we observed less variation in AIC and HIV prevalence, with all model CIs overlapping with that of the IPW model. For both sexes, the Student's‐*t* copula had the best fit and was thus used for the main analysis. Copula tests for all countries are shown in Figure S2.

Prevalence estimates for men were higher in selection models than the IPW model in Tanzania (7.9% vs. 3.8%), Uganda (4.7% vs. 4.2%), Zimbabwe (13.5% vs. 12.0%), Lesotho (24.3% vs. 19.3%) and Eswatini (20.8% vs. 19.9%); and for women in Tanzania (7.9% vs. 6.2%), Zambia (15.5% vs. 14.3%), Zimbabwe (18.1% vs. 15.9%), Lesotho (34.9% vs. 29.7%) and Eswatini (37.5% vs. 34.3%). The differential between IPW and selection model point estimates ranged between –0.8% and 5.2%. Compared with IPW models, selection models generally yielded lower estimated female‐to‐male prevalence ratios; the largest change was observed in Tanzania where the female‐to‐male prevalence ratio disappeared from 1.63 to 1.00 (Table 2).

Sensitivity analyses restricted to participants who completed an individual interview are shown in Table S4 and Figure S3. The direction and magnitude of selection bias estimated by the selection model were inconsistent with analyses using all eligible household members. Notably, however, the selection model estimates were typically higher than the IPW estimate in these analyses as well, except for Malawi and Zambia for men and Tanzania for women.

## 4 DISCUSSION

In this study, we assessed the sensitivity of national HIV prevalence estimates to the assumptions made about test non‐participation using data from seven population‐based HIV surveys in sub‐Saharan Africa. Compared to unadjusted estimates, standard methods to account for non‐response under MAR assumptions (IPW and MI) produced only small changes in estimated prevalence in most countries. However, using selection models—a method that relaxes the assumption that non‐participation was MAR conditional on observed characteristics—led to increased prevalence estimates compared to IPW models in several cases where MI did not.

Our study replicates and extends a previous analysis using older DHS data from 12 countries, five of which were also included here (Malawi, Zambia, Eswatini, Zimbabwe and Lesotho) [16]. The impact of using selection models in our analysis was less extreme than in the earlier work, but we found that selection model estimates deviated from IPW and MI models in a greater proportion of surveys than Hogan et al. did [16]. This may reflect changes over time in the strength of association between HIV status and willingness to participate, perhaps because of reductions in HIV‐related stigma due to increased availability of treatment [35]. Alternatively, the more modest differences may reflect methodological improvements, including the ability to model different forms of the relationship between selection and outcome via copula functions, and improved convergence properties via the incorporation of ridge penalties in model estimation [12, 34].

Selection models potentially provide more accurate measures of an outcome when the assumptions of commonly used approaches are inappropriate. When study non‐participation is driven by factors not observable to implementers, IPW or MI methods operate under incorrect assumptions regarding the mechanisms that determine missingness. IPW models can also underestimate uncertainty due to missing data in variance estimation. The selection model CIs were often, but not always, wider than those of naïve and IPW models, reflecting uncertainty in both sampling variability and regression parameters, similar to results seen in prior studies [16]. Some selection model estimates did not have wider CIs, though, possibly due to better fit achieved using the specified copula function and increased efficiency via joint regression modelling procedures [12, 16]. However, even in cases where the selection model estimates were less precise, results were nonetheless statistically significantly and meaningfully different to IPW estimates in several cases. In all such cases here, when multiple copulae diverged substantially from the IPW estimate, the estimates from these copulae models were similar to one another. However, such accord may not always be the case and subject matter knowledge should be used to guide whether the selection model estimates are plausible.

The wide variability seen in estimates across different copulae suggests that selection models are in fact sensitive to this model specification. Indeed, copula selection proved to be non‐trivial in several surveys where multiple copulae yielded divergent HIV prevalence estimates. As the example of Tanzania illustrated above, findings suggested that a negatively correlated copulae (Student's‐*t*) best represented the relationship between selection and outcome, meaning that HIV‐positive status was associated with lower likelihood of participation. Furthermore, Student's‐*t* is a symmetric copula, indicating that the association was driven equally by lower participation among HIV‐positive and higher participation among HIV‐negative individuals. In contrast, teardrop‐shaped copulae suggest that the association is stronger for certain individuals; for example, the Joe 90 rotation copula selected in Lesotho shows that the negative association between HIV status and participation is driven more by individuals who are HIV negative (Figure S4).

We cannot rule out misspecification of the selection model equations. Our selection model results for men in Tanzania yielded a two‐fold increase in HIV prevalence compared to IPW estimates despite high participation rates (84%). This suggests a degree of selection bias where approximately one‐third of men who refused participation were HIV positive (compared to 3.8% among participants). The estimated correlation between selection and outcome model errors was strong (Kendall's tau: –0.548, Figure S4); however, validation requires external knowledge of study design and data collection. Similarly, MI estimates for men in Malawi and Zambia drastically diverged from the IPW models. These findings were not replicated among individual interview participants only (Figure S3), suggesting that the selection biases likely stemmed from household non‐participation. Male heads of household may disproportionately refuse when HIV positive, but this hypothesis is untestable with the available data. Future surveys should collect more robust household‐level predictors of participation and HIV risk.

The validity of our selection model findings rests centrally on whether interviewer identity is a valid selection variable (i.e. it is unrelated to HIV status conditional on observed variables). This assumption is conceptually untestable. Interviewers in PHIA were intentionally matched to geographic areas based on language. However, other unplanned matching may have occurred, for example if high‐performing interviewers were assigned to difficult areas, or to participants by sex or age. If unplanned matching occurred and was strongly correlated with HIV status, the exclusion criterion would be violated, although its impact on our findings cannot be discerned. Future studies could limit such violations by randomizing interviewer assignment *a priori*. Randomized assignment of interviewers within defined geographic areas has previously been shown to be feasible and improve identification of interviewer effects without substantial detriment to overall survey quality [36]. We are also unable to determine whether the elicitation of blood test consent prior to the interview in Eswatini (whereas it was elicited after the interview in all other countries) affected likelihood of consent and thus the selection model results. It is, for example, possible that interviewers develop rapport during the interview, increasing the likelihood of blood test participation. However, we found no stronger evidence of selection bias in Eswatini than in the other countries. Lastly, this analysis cannot account for unobserved predictors of household non‐participation.

## 5 CONCLUSIONS

In this study, we demonstrate the utility of selection models as a valuable part of triangulation in estimating HIV prevalence [37]. While selection models require additional assumptions and careful assessment of model sensitivity, they can evaluate the potential impact of non‐participation biases compared to other analytical methods where non‐response is high, missingness is likely non‐random conditional on observed characteristics and a plausible selection variable is available. In the context of population‐based studies, this includes biomarker and other objectively verifiable outcomes, particularly those subject to stigma or fear‐based perceptions (e.g. viral load for measuring treatment adherence). Selection models also enable exploration of the relationships between study participation and outcome, which is itself of scientific interest. Given the accessibility of flexible tools in readily available software, selection models can be easily implemented and have the potential to improve modelling and inference in survey analysis. Furthermore, selection model results may highlight areas for improving future surveys through enhanced interviewer training and supervision and targeted recruitment of sub‐populations with known non‐participation biases observed in previous surveys.

## COMPETING INTERESTS

The authors declare that they have no competing interests.

## AUTHORS’ CONTRIBUTIONS

GH and AMP conceptualized this project. AMP conducted the analyses and wrote the first draft of the paper, with support from GH and GM. All authors contributed to the study design, data interpretation and final revisions to the text and have approved the final version.

## DISCLAIMER

The contents of this manuscript are solely the responsibility of the authors and do not necessarily represent the official views of the Centers for Disease Control and Prevention or the National Institutes of Health.

## Supporting information


**Figure S1**: Consent rates and HIV prevalence by interviewer
**Figure S2**: Selection model results by copula
**Figure S3**: HIV prevalence estimates among adults aged 15–49 under different missingness assumptions using data from individual interview participants only
**Figure S4**: Copula contour plots
**Table S1**: Model specifications
**Table S2a**: Sample characteristics for Tanzania
**Table S2b**: Sample characteristics for Uganda
**Table S2c**: Sample characteristics for Malawi
**Table S2d**: Sample characteristics for Zambia
**Table S2e**: Sample characteristics for Zimbabwe
**Table S2f**: Sample characteristics for Lesotho
**Table S2g**: Sample characteristics for Eswatini
**Table S3**: Interviewer‐level participation rate (%) and HIV test prevalence (%)
**Table S4**: HIV prevalence estimates among adults aged 15–49 under different missingness assumptions using data from individual interview participants only

## Data Availability

The data used in this manuscript are publically available from the Population‐based HIV Impact Assessment (PHIA) Project website at https://phia‐data.icap.columbia.edu/
