# Supplementary material for: Correcting for selection bias in HIV prevalence estimates: an application of sample selection models using data from population‐based HIV surveys in seven sub‐Saharan African countries
Source: J Int AIDS Soc. 2022 Aug 5;25(8):e25954. doi: 10.1002/jia2.25954 (PMC9353488; doi:10.1002/jia2.25954)
Supplement: Supplementary file 1 — Figure S1: Consent rates and HIV prevalence by interviewer Figure S2: Selection model results by copula Figure S3: HIV prevalence estimates among adults aged 15–49 under different missingness assumptions using data from individual interview participants only Figure S4: Copula contour plots Table S1: Model specifications Table S2a: Sample characteristics for Tanzania Table S2b: Sample characteristics for Uganda Table S2c: Sample characteristics for Malawi Table S2d: Sample characteristics for Zambia Table S2e: Sample characteristics for Zimbabwe Table S2f: Sample characteristics for Lesotho Table S2g: Sample characteristics for Eswatini Table S3: Interviewer‐level participation rate (%) and HIV test prevalence (%) Table S4: HIV prevalence estimates among adults aged 15–49 under different missingness assumptions using data from individual interview participants only [file JIA2-25-e25954-s001.docx]

**Title:** Correcting for selection bias in HIV prevalence estimates: an application of sample selection models using data from population-based HIV surveys in seven sub-Saharan African countries

# Supplementary Materials

**Supplementary Table 1. Model specifications**

|  | **Model** | | | | | |
| --- | --- | --- | --- | --- | --- | --- |
|  | **Naive** | **IPW** | **MI - imputation model** | **MI - outcome model** | **Selection model - selection equation** | **Selection model - outcome equation** |
| **Outcome variable** | HIV status | HIV status | Various^a^ | HIV status | HIV test participation | HIV status |
| **Variables** |  |  |  |  |  |  |
| Household-level |  |  |  |  |  |  |
| Region/province |  | X | X | X | X^b^ | X^b^ |
| Urban/rural |  | X | X | X | X | X |
| HH wealth quintile |  | X | X | X | X | X |
| Individual-level |  |  |  |  |  |  |
| Age |  | X | X | X | X | X |
| Education |  | X | X | X | X | X |
| Ethnicity |  | X | X | X | X | X |
| Marital status |  | X | X | X | X | X |
| Ever had sex |  | X | X | X | X | X |
| Ever tested for HIV |  | X | X | X | X | X |
| Circumcised |  | X | X | X | X | X |
| Pregnancy status |  | X | X | X | X | X |
| On antiretroviral therapy |  | X | X | X | X | X |
| Language |  |  |  |  | X | X |
| Interviewer identity |  |  |  |  | X |  |
| **Analytic weights** |  |  |  |  |  |  |
| Design weights | X | X | X | X | X | X |
| Household non-participation weights | X | X | X | X | X | X |
| Blood test participation weights (inverse probability) |  |  | X |  |  |  |
| Abbreviations: IPW: inverse probability weighting, MI: multiple imputation.  ^a^ MI was performed using chained equations, thus each variable with missing data was sequentially modeled as the outcome for imputation. Predictor variables included were the same across all variables for simplicity.  ^b^ Region/province were included in selection model selection and outcome equations using a Markov random field smoother to improve convergence properties in generalized joint regression modeling procedure. | | | | | | |

**Supplementary Table 2a. Sample characteristics for Tanzania**

|  | **Males** | | | | | | **Females** | | | | | |
| --- | --- | --- | --- | --- | --- | --- | --- | --- | --- | --- | --- | --- |
|  | **All eligible household members** | | **Interview participants** | | **Blood test participants** | | **All eligible household members** | | **Interview participants** | | **Blood test participants** | |
| **Variable** | **%** | **N** | **%** | **N** | **%** | **N** | **%** | **N** | **%** | **N** | **%** | **N** |
| Geographic area (Region) |  |  |  |  |  |  |  |  |  |  |  |  |
| Dodoma | 1.5 | 200 | 1.5 | 171 | 1.4 | 154 | 1.5 | 241 | 1.5 | 221 | 1.4 | 204 |
| Arusha | 1.5 | 195 | 1.4 | 156 | 1.3 | 139 | 1.7 | 278 | 1.6 | 243 | 1.5 | 218 |
| Kilimanjaro | 1.7 | 217 | 1.6 | 180 | 1.4 | 159 | 1.7 | 273 | 1.7 | 257 | 1.6 | 236 |
| Tanga | 1.8 | 231 | 1.8 | 213 | 1.9 | 204 | 1.9 | 300 | 1.9 | 288 | 1.9 | 281 |
| Morogoro | 2.3 | 297 | 2.3 | 260 | 2.3 | 247 | 2.7 | 427 | 2.7 | 413 | 2.7 | 391 |
| Pwani | 5.6 | 726 | 5.4 | 622 | 5.2 | 573 | 6.0 | 962 | 6.0 | 919 | 5.8 | 852 |
| Dar es Salaam | 6.5 | 855 | 6.1 | 700 | 5.6 | 619 | 6.7 | 1079 | 6.6 | 1004 | 6.4 | 939 |
| Lindi | 1.0 | 137 | 1.1 | 131 | 1.2 | 127 | 0.9 | 143 | 0.9 | 135 | 0.9 | 132 |
| Mtwara | 1.3 | 171 | 1.4 | 156 | 1.3 | 142 | 1.1 | 172 | 1.1 | 166 | 1.1 | 156 |
| Ruvuma | 5.8 | 757 | 6.0 | 696 | 6.0 | 657 | 5.4 | 863 | 5.4 | 828 | 5.5 | 803 |
| Iringa | 4.2 | 545 | 4.1 | 473 | 4.0 | 443 | 4.2 | 675 | 4.2 | 645 | 4.2 | 617 |
| Mbeya | 4.3 | 561 | 4.2 | 490 | 4.1 | 450 | 4.5 | 727 | 4.5 | 690 | 4.3 | 634 |
| Songwe | 1.0 | 137 | 0.9 | 108 | 0.9 | 100 | 1.1 | 174 | 1.0 | 159 | 1.0 | 146 |
| Singida | 10.4 | 1357 | 10.5 | 1208 | 10.8 | 1182 | 9.6 | 1544 | 9.2 | 1409 | 9.4 | 1373 |
| Tabora | 6.7 | 874 | 6.7 | 770 | 6.8 | 747 | 6.9 | 1110 | 6.9 | 1055 | 7.0 | 1023 |
| Rukwa | 2.2 | 282 | 2.3 | 260 | 2.4 | 258 | 2.2 | 358 | 2.3 | 347 | 2.4 | 344 |
| Kigoma | 7.3 | 947 | 7.5 | 860 | 7.7 | 840 | 6.6 | 1066 | 6.7 | 1021 | 6.9 | 1005 |
| Shinyanga | 2.7 | 357 | 2.9 | 333 | 3.0 | 324 | 2.4 | 388 | 2.4 | 372 | 2.5 | 367 |
| Kagera | 4.0 | 521 | 4.1 | 478 | 4.2 | 459 | 3.8 | 616 | 3.9 | 593 | 3.9 | 572 |
| Mwanza | 2.4 | 319 | 2.5 | 289 | 2.6 | 285 | 2.7 | 432 | 2.7 | 416 | 2.8 | 407 |
| Mara | 1.5 | 193 | 1.5 | 169 | 1.5 | 160 | 1.4 | 221 | 1.4 | 215 | 1.4 | 205 |
| Manyara | 2.5 | 321 | 2.4 | 279 | 2.4 | 267 | 2.8 | 452 | 2.9 | 437 | 2.8 | 409 |
| Njombe | 7.6 | 995 | 7.6 | 878 | 7.8 | 854 | 7.3 | 1171 | 7.5 | 1135 | 7.6 | 1119 |
| Katavi | 2.1 | 272 | 2.2 | 252 | 2.2 | 245 | 2.4 | 382 | 2.4 | 364 | 2.5 | 362 |
| Simiyu | 2.7 | 353 | 2.8 | 326 | 2.9 | 319 | 2.5 | 403 | 2.6 | 394 | 2.7 | 388 |
| Geita | 4.2 | 546 | 4.3 | 491 | 4.3 | 468 | 4.4 | 700 | 4.3 | 659 | 4.4 | 639 |
| Kaskazini Unguja | 0.8 | 107 | 0.8 | 90 | 0.8 | 87 | 0.9 | 147 | 0.9 | 140 | 0.9 | 136 |
| Kusini Unguja | 0.7 | 94 | 0.7 | 84 | 0.8 | 83 | 0.7 | 107 | 0.7 | 102 | 0.7 | 99 |
| Mjini Magharibi | 2.4 | 319 | 2.2 | 259 | 2.2 | 244 | 2.5 | 394 | 2.4 | 368 | 2.4 | 350 |
| Kaskazini Pemba | 0.6 | 72 | 0.5 | 62 | 0.5 | 54 | 0.7 | 109 | 0.7 | 101 | 0.6 | 91 |
| Kusini Pemba | 0.8 | 101 | 0.8 | 87 | 0.7 | 81 | 0.9 | 143 | 0.9 | 138 | 0.9 | 131 |
| Urbanicity |  |  |  |  |  |  |  |  |  |  |  |  |
| Urban | 33.8 | 4413 | 32.3 | 3728 | 31.3 | 3435 | 35.7 | 5739 | 35.9 | 5473 | 35.4 | 5174 |
| Rural | 66.2 | 8646 | 67.7 | 7803 | 68.7 | 7536 | 64.3 | 10318 | 64.1 | 9761 | 64.6 | 9455 |
| Wealth quintile |  |  |  |  |  |  |  |  |  |  |  |  |
| Lowest | 17.9 | 2341 | 17.0 | 1957 | 16.2 | 1777 | 19.1 | 3071 | 18.9 | 2886 | 18.6 | 2715 |
| Second | 18.7 | 2443 | 18.5 | 2138 | 18.1 | 1989 | 18.9 | 3031 | 19.2 | 2929 | 19.0 | 2774 |
| Middle | 21.5 | 2814 | 21.8 | 2513 | 22.0 | 2417 | 21.8 | 3502 | 22.1 | 3371 | 22.2 | 3246 |
| Fourth | 20.6 | 2684 | 21.3 | 2457 | 21.8 | 2391 | 19.9 | 3193 | 19.9 | 3027 | 20.2 | 2948 |
| Highest | 21.2 | 2774 | 21.4 | 2464 | 21.8 | 2395 | 20.3 | 3255 | 19.8 | 3016 | 20.1 | 2941 |
| Missing | 0 | 3 | 0 | 2 | 0 | 2 | 0 | 5 | 0 | 5 | 0 | 5 |
| Age group |  |  |  |  |  |  |  |  |  |  |  |  |
| 15-19 | 22.7 | 2958 | 22.8 | 2624 | 23.1 | 2533 | 20.5 | 3284 | 20.2 | 3073 | 20.5 | 2999 |
| 20-24 | 17.8 | 2321 | 18.1 | 2082 | 18.1 | 1987 | 19.3 | 3106 | 19.4 | 2959 | 19.4 | 2845 |
| 25-29 | 15.6 | 2032 | 15.4 | 1779 | 15.2 | 1670 | 17.4 | 2797 | 17.5 | 2660 | 17.2 | 2521 |
| 30-34 | 13.6 | 1773 | 13.4 | 1541 | 13.2 | 1453 | 14.1 | 2264 | 14.2 | 2158 | 14.1 | 2062 |
| 35-39 | 12.1 | 1581 | 11.9 | 1373 | 11.9 | 1307 | 11.8 | 1897 | 12.0 | 1828 | 12.0 | 1749 |
| 40-45 | 10.5 | 1372 | 10.6 | 1217 | 10.4 | 1144 | 9.7 | 1552 | 9.6 | 1466 | 9.6 | 1405 |
| 45-49 | 7.8 | 1022 | 7.9 | 915 | 8.0 | 877 | 7.2 | 1157 | 7.2 | 1090 | 7.2 | 1048 |
| Education level |  |  |  |  |  |  |  |  |  |  |  |  |
| No education | 10.5 | 1215 | 10.5 | 1215 | 10.6 | 1166 | 16.5 | 2513 | 16.5 | 2513 | 16.7 | 2439 |
| Primary | 61.9 | 7132 | 61.9 | 7132 | 62.5 | 6856 | 60.2 | 9159 | 60.1 | 9159 | 60.4 | 8838 |
| Secondary | 25.1 | 2896 | 25.1 | 2896 | 24.7 | 2708 | 22.3 | 3389 | 22.2 | 3389 | 21.9 | 3202 |
| More than secondary | 2.5 | 284 | 2.5 | 284 | 2.2 | 238 | 1.1 | 165 | 1.1 | 165 | 1.0 | 142 |
| Missing |  |  | 0 | 4 | 0 | 3 |  |  | 0.1 | 8 | 0.1 | 8 |
| Marital status |  |  |  |  |  |  |  |  |  |  |  |  |
| Never married | 39.6 | 4557 | 39.5 | 4557 | 39.5 | 4330 | 24.2 | 3672 | 24.1 | 3672 | 24 | 3517 |
| Married or living together | 54.4 | 6257 | 54.3 | 6257 | 54.3 | 5954 | 62.4 | 9486 | 62.3 | 9486 | 62.3 | 9117 |
| Divorced or separated | 5.5 | 630 | 5.5 | 630 | 5.5 | 603 | 10.4 | 1588 | 10.4 | 1588 | 10.4 | 1527 |
| Widowed | 0.5 | 57 | 0.5 | 57 | 0.5 | 57 | 3.0 | 458 | 3.0 | 458 | 3.0 | 441 |
| Missing |  |  | 0.3 | 30 | 0.2 | 27 |  |  | 0.2 | 30 | 0.2 | 27 |
| Circumcised |  |  |  |  |  |  |  |  |  |  |  |  |
| No | 47.9 | 5378 | 46.6 | 5378 | 47.2 | 5179 |  |  |  |  |  |  |
| Yes | 52.1 | 5849 | 50.7 | 5849 | 50.2 | 5512 |  |  |  |  |  |  |
| Missing |  |  | 2.6 | 304 | 2.6 | 280 |  |  |  |  |  |  |
| Pregnant |  |  |  |  |  |  |  |  |  |  |  |  |
| No |  |  |  |  |  |  | 90.9 | 13618 | 89.4 | 13618 | 89.4 | 13085 |
| Yes |  |  |  |  |  |  | 9.1 | 1358 | 8.9 | 1358 | 8.9 | 1302 |
| Missing |  |  |  |  |  |  |  |  | 1.7 | 258 | 1.7 | 242 |
| Ever tested for HIV |  |  |  |  |  |  |  |  |  |  |  |  |
| No | 36.1 | 4151 | 36 | 4151 | 36.4 | 3993 | 20.7 | 3140 | 20.6 | 3140 | 21 | 3066 |
| Yes | 63.9 | 7354 | 63.8 | 7354 | 63.4 | 6952 | 79.3 | 12063 | 79.2 | 12063 | 78.9 | 11535 |
| Missing |  |  | 0.2 | 26 | 0.2 | 26 |  |  | 0.2 | 31 | 0.2 | 28 |
| Ever had sex |  |  |  |  |  |  |  |  |  |  |  |  |
| No | 14.9 | 1948 | 16.9 | 1948 | 16.9 | 1852 | 11.4 | 1730 | 11.4 | 1730 | 11.4 | 1664 |
| Yes | 73.4 | 9583 | 83.1 | 9583 | 83.1 | 9119 | 88.6 | 13504 | 88.6 | 13504 | 88.6 | 12965 |
| Missing | 11.7 | 1528 | 0 | 0 | 0 | 0 |  |  | 0 | 0 | 0 | 0 |

**Supplementary Table 2b. Sample characteristics for Uganda**

|  | **Males** | | | | | | **Females** | | | | | |
| --- | --- | --- | --- | --- | --- | --- | --- | --- | --- | --- | --- | --- |
|  | **All eligible household members** | | **Interview participants** | | **Blood test participants** | | **All eligible household members** | | **Interview participants** | | **Blood test participants** | |
| **Variable** | **%** | **N** | **%** | **N** | **%** | **N** | **%** | **N** | **%** | **N** | **%** | **N** |
| Geographic area (Region) |  |  |  |  |  |  |  |  |  |  |  |  |
| Kampala | 9.4 | 1109 | 9.2 | 1017 | 9.3 | 1005 | 9.5 | 1443 | 9.5 | 1411 | 9.5 | 1393 |
| Central1 | 7.3 | 857 | 7.3 | 809 | 7.3 | 793 | 7.7 | 1167 | 7.7 | 1151 | 7.7 | 1140 |
| Central2 | 8.1 | 951 | 7.7 | 844 | 7.6 | 827 | 8.7 | 1329 | 8.7 | 1290 | 8.6 | 1271 |
| East Central | 10.1 | 1193 | 10.2 | 1126 | 10.1 | 1093 | 10.4 | 1577 | 10.4 | 1544 | 10.4 | 1526 |
| Eastern | 14.3 | 1687 | 14.2 | 1561 | 14.2 | 1536 | 14.4 | 2191 | 14.3 | 2128 | 14.3 | 2105 |
| Karamoja | 12.4 | 1458 | 12.5 | 1383 | 12.6 | 1370 | 12.0 | 1821 | 12.0 | 1782 | 12.0 | 1769 |
| Northern | 13.4 | 1576 | 13.2 | 1455 | 13.2 | 1437 | 13.9 | 2115 | 13.9 | 2063 | 13.9 | 2048 |
| West Nile | 9.0 | 1064 | 9.2 | 1012 | 9.1 | 989 | 7.6 | 1154 | 7.6 | 1129 | 7.6 | 1115 |
| Western | 9.0 | 1059 | 9.2 | 1009 | 9.2 | 1001 | 8.3 | 1262 | 8.3 | 1238 | 8.3 | 1227 |
| South Western | 7.1 | 834 | 7.3 | 805 | 7.4 | 803 | 7.5 | 1141 | 7.6 | 1124 | 7.6 | 1122 |
| Urbanicity |  |  |  |  |  |  |  |  |  |  |  |  |
| Urban | 27.2 | 3201 | 26.3 | 2900 | 26.2 | 2849 | 29.2 | 4441 | 29.1 | 4322 | 29.1 | 4276 |
| Rural | 72.8 | 8587 | 73.7 | 8121 | 73.8 | 8005 | 70.8 | 10759 | 70.9 | 10538 | 70.9 | 10440 |
| Wealth quintile |  |  |  |  |  |  |  |  |  |  |  |  |
| Lowest | 27.5 | 3238 | 27.8 | 3059 | 27.8 | 3013 | 26.5 | 4023 | 26.5 | 3934 | 26.5 | 3893 |
| Second | 20.1 | 2375 | 20.5 | 2256 | 20.5 | 2227 | 18.8 | 2856 | 18.8 | 2793 | 18.9 | 2774 |
| Middle | 18.0 | 2120 | 18.2 | 2002 | 18.1 | 1970 | 18.3 | 2786 | 18.4 | 2731 | 18.4 | 2704 |
| Fourth | 16.2 | 1909 | 16.1 | 1771 | 16.1 | 1745 | 16.7 | 2532 | 16.6 | 2474 | 16.6 | 2449 |
| Highest | 18.2 | 2145 | 17.5 | 1933 | 17.5 | 1899 | 19.8 | 3003 | 19.7 | 2928 | 19.7 | 2896 |
| Missing | 0 | 1 | 0 | 0 | 0 | 0 | 0 | 0 | 0 | 0 | 0 | 0 |
| Age group |  |  |  |  |  |  |  |  |  |  |  |  |
| 15-19 | 25.9 | 3050 | 26.1 | 2872 | 26.1 | 2834 | 22.6 | 3433 | 22.3 | 3316 | 22.3 | 3289 |
| 20-24 | 19.2 | 2265 | 19.0 | 2098 | 19.0 | 2063 | 20.9 | 3184 | 20.8 | 3097 | 20.8 | 3059 |
| 25-29 | 15.2 | 1794 | 15.2 | 1670 | 15.1 | 1637 | 17.4 | 2644 | 17.5 | 2606 | 17.5 | 2574 |
| 30-34 | 12.8 | 1508 | 12.7 | 1397 | 12.7 | 1380 | 13.6 | 2072 | 13.7 | 2040 | 13.8 | 2024 |
| 35-39 | 10.6 | 1244 | 10.6 | 1167 | 10.5 | 1143 | 10.7 | 1625 | 10.7 | 1590 | 10.7 | 1573 |
| 40-45 | 8.6 | 1016 | 8.7 | 959 | 8.8 | 952 | 8.2 | 1239 | 8.2 | 1225 | 8.2 | 1214 |
| 45-49 | 7.7 | 911 | 7.8 | 858 | 7.8 | 845 | 6.6 | 1003 | 6.6 | 986 | 6.7 | 983 |
| Education level |  |  |  |  |  |  |  |  |  |  |  |  |
| No education | 43.7 | 4785 | 43.4 | 4785 | 43.5 | 4717 | 53.9 | 7961 | 53.6 | 7961 | 53.6 | 7885 |
| Primary | 43.0 | 4715 | 42.8 | 4715 | 42.7 | 4637 | 37.8 | 5592 | 37.6 | 5592 | 37.7 | 5544 |
| Secondary | 13.3 | 1454 | 13.2 | 1454 | 13.2 | 1433 | 8.3 | 1229 | 8.3 | 1229 | 8.2 | 1209 |
| Missing |  |  | 0.6 | 67 | 0.6 | 67 |  |  | 0.5 | 78 | 0.5 | 78 |
| Ethnicity |  |  |  |  |  |  |  |  |  |  |  |  |
| Baganda | 13.7 | 1619 | 14.7 | 1619 | 14.6 | 1581 | 15.4 | 2335 | 15.7 | 2335 | 15.6 | 2303 |
| Bankyakore | 6.9 | 814 | 7.4 | 814 | 7.5 | 810 | 7.9 | 1194 | 8.0 | 1194 | 8.1 | 1188 |
| Basoga | 8.2 | 968 | 8.8 | 968 | 8.7 | 943 | 8.5 | 1293 | 8.7 | 1293 | 8.7 | 1280 |
| Bakiga | 3.6 | 421 | 3.8 | 421 | 3.9 | 419 | 3.8 | 580 | 3.9 | 580 | 3.9 | 576 |
| Iteso | 9.9 | 1162 | 10.5 | 1162 | 10.6 | 1152 | 9.4 | 1426 | 9.6 | 1426 | 9.6 | 1415 |
| Langi | 6.0 | 711 | 6.5 | 711 | 6.5 | 701 | 4.8 | 733 | 4.9 | 733 | 4.9 | 726 |
| Bagisu/Sabiny | 7.6 | 892 | 8.1 | 892 | 8.1 | 884 | 7.7 | 1171 | 7.9 | 1171 | 7.9 | 1164 |
| Acholi | 3.1 | 364 | 3.3 | 364 | 3.2 | 351 | 3.0 | 460 | 3.1 | 460 | 3.1 | 454 |
| Lugbara/Madi | 8.3 | 977 | 8.9 | 977 | 8.9 | 964 | 9.5 | 1447 | 9.7 | 1447 | 9.7 | 1432 |
| Batoro | 2.2 | 255 | 2.3 | 255 | 2.3 | 251 | 2.1 | 323 | 2.2 | 323 | 2.2 | 320 |
| Banyoro | 3.0 | 353 | 3.2 | 353 | 3.2 | 352 | 2.8 | 423 | 2.8 | 423 | 2.9 | 422 |
| Others | 21.0 | 2479 | 22.5 | 2479 | 22.5 | 2440 | 22.8 | 3468 | 23.3 | 3468 | 23.3 | 3429 |
| Missing | 6.6 | 773 | 0.1 | 6 | 0.1 | 6 | 2.3 | 347 | 0 | 7 | 0 | 7 |
| Marital status |  |  |  |  |  |  |  |  |  |  |  |  |
| Never married | 41.7 | 4588 | 41.6 | 4588 | 41.6 | 4515 | 25.3 | 3750 | 25.2 | 3750 | 25.2 | 3709 |
| Married or living together | 50.8 | 5586 | 50.7 | 5586 | 50.7 | 5505 | 58.7 | 8697 | 58.5 | 8697 | 58.5 | 8610 |
| Divorced or separated | 7.2 | 789 | 7.2 | 789 | 7.2 | 777 | 12.8 | 1899 | 12.8 | 1899 | 12.8 | 1887 |
| Widowed | 0.4 | 39 | 0.4 | 39 | 0.4 | 39 | 3.1 | 466 | 3.1 | 466 | 3.1 | 463 |
| Missing |  |  | 0.2 | 19 | 0.2 | 18 |  |  | 0.3 | 48 | 0.3 | 47 |
| Circumcised |  |  |  |  |  |  |  |  |  |  |  |  |
| No | 77.6 | 8291 | 75.2 | 8291 | 75.4 | 8180 |  |  |  |  |  |  |
| Yes | 22.4 | 2396 | 21.7 | 2396 | 21.7 | 2355 |  |  |  |  |  |  |
| Missing |  |  | 3 | 334 | 2.9 | 319 |  |  |  |  |  |  |
| Pregnant |  |  |  |  |  |  |  |  |  |  |  |  |
| No |  |  |  |  |  |  | 90.3 | 13200 | 88.8 | 13200 | 88.8 | 13074 |
| Yes |  |  |  |  |  |  | 9.7 | 1418 | 9.5 | 1418 | 9.5 | 1401 |
| Missing |  |  |  |  |  |  |  |  | 1.6 | 242 | 1.6 | 241 |
| Ever tested for HIV |  |  |  |  |  |  |  |  |  |  |  |  |
| No | 30.6 | 3368 | 30.6 | 3368 | 30.6 | 3325 | 15.4 | 2286 | 15.4 | 2286 | 15.4 | 2263 |
| Yes | 69.4 | 7640 | 69.3 | 7640 | 69.3 | 7517 | 84.6 | 12552 | 84.5 | 12552 | 84.5 | 12431 |
| Missing |  |  | 0.1 | 13 | 0.1 | 12 |  |  | 0.1 | 22 | 0.1 | 22 |
| Ever had sex |  |  |  |  |  |  |  |  |  |  |  |  |
| No | 16.3 | 1787 | 16.2 | 1787 | 16.2 | 1761 | 12.5 | 1860 | 12.5 | 1860 | 12.5 | 1844 |
| Yes | 83.7 | 9207 | 83.5 | 9207 | 83.6 | 9070 | 87.5 | 12993 | 87.4 | 12993 | 87.4 | 12865 |
| Missing |  |  | 0.2 | 27 | 0.2 | 23 |  |  | 0 | 7 | 0 | 7 |

**Supplementary Table 2c. Sample characteristics for Malawi**

|  | **Males** | | | | | | **Females** | | | | | |
| --- | --- | --- | --- | --- | --- | --- | --- | --- | --- | --- | --- | --- |
|  | **All eligible household members** | | **Interview participants** | | **Blood test participants** | | **All eligible household members** | | **Interview participants** | | **Blood test participants** | |
| **Variable** | **%** | **N** | **%** | **N** | **%** | **N** | **%** | **N** | **%** | **N** | **%** | **N** |
| Geographic area (Zone) |  |  |  |  |  |  |  |  |  |  |  |  |
| North | 12.8 | 1154 | 13.4 | 976 | 14.2 | 894 | 12.1 | 1337 | 12.4 | 1264 | 12.9 | 1157 |
| Central-East | 14.1 | 1270 | 15.0 | 1090 | 15.7 | 987 | 12.8 | 1414 | 13.1 | 1340 | 13.5 | 1207 |
| Central-West | 10.7 | 966 | 11.2 | 814 | 11.7 | 735 | 11.3 | 1246 | 11.3 | 1151 | 11.3 | 1009 |
| Lilongwe City | 19.5 | 1763 | 18.4 | 1340 | 18.4 | 1159 | 17.9 | 1971 | 17.6 | 1795 | 18.0 | 1607 |
| South-East | 10.7 | 966 | 10.7 | 779 | 9.9 | 627 | 12.7 | 1399 | 12.6 | 1288 | 11.7 | 1051 |
| South-West | 14.1 | 1272 | 14.4 | 1047 | 14.3 | 900 | 16.3 | 1789 | 16.5 | 1687 | 16.4 | 1472 |
| Blantyre City | 18.2 | 1639 | 17.0 | 1237 | 15.9 | 1004 | 16.8 | 1850 | 16.4 | 1678 | 16.2 | 1446 |
| Urbanicity |  |  |  |  |  |  |  |  |  |  |  |  |
| Urban | 42.2 | 3808 | 39.7 | 2891 | 38.7 | 2439 | 39.2 | 4312 | 38.5 | 3926 | 38.8 | 3470 |
| Rural | 57.8 | 5222 | 60.3 | 4392 | 61.3 | 3867 | 60.8 | 6694 | 61.5 | 6277 | 61.2 | 5479 |
| Wealth quintile |  |  |  |  |  |  |  |  |  |  |  |  |
| Lowest | 10.4 | 940 | 10.6 | 770 | 10.7 | 673 | 12.3 | 1349 | 12.4 | 1269 | 12.0 | 1070 |
| Second | 13.2 | 1193 | 14.2 | 1034 | 14.4 | 907 | 13.8 | 1523 | 14.0 | 1430 | 13.9 | 1245 |
| Middle | 14.9 | 1344 | 15.5 | 1127 | 15.6 | 985 | 15.6 | 1712 | 15.8 | 1607 | 15.9 | 1426 |
| Fourth | 20.7 | 1867 | 20.8 | 1514 | 21.4 | 1352 | 19.6 | 2158 | 19.9 | 2030 | 20.3 | 1815 |
| Highest | 40.8 | 3684 | 39.0 | 2838 | 37.9 | 2389 | 38.7 | 4263 | 37.9 | 3867 | 37.9 | 3393 |
| Missing | 0 | 2 | 0 | 0 | 0 | 0 | 0 | 1 | 0 | 0 | 0 | 0 |
| Age group |  |  |  |  |  |  |  |  |  |  |  |  |
| 15-19 | 23.7 | 2144 | 23.1 | 1680 | 23.7 | 1497 | 19.9 | 2186 | 18.2 | 1858 | 18.4 | 1646 |
| 20-24 | 18.3 | 1654 | 19.1 | 1389 | 18.7 | 1181 | 21.9 | 2408 | 22.0 | 2244 | 21.6 | 1934 |
| 25-29 | 15.7 | 1420 | 15.4 | 1120 | 14.9 | 938 | 16.8 | 1854 | 17.2 | 1756 | 16.9 | 1511 |
| 30-34 | 14.2 | 1283 | 13.9 | 1010 | 13.8 | 870 | 15.3 | 1686 | 15.8 | 1608 | 15.9 | 1425 |
| 35-39 | 12.2 | 1106 | 12.0 | 877 | 12.1 | 765 | 11.9 | 1305 | 12.1 | 1238 | 12.3 | 1097 |
| 40-45 | 9.3 | 840 | 9.7 | 706 | 9.7 | 609 | 8.3 | 908 | 8.6 | 873 | 8.8 | 785 |
| 45-49 | 6.5 | 583 | 6.9 | 501 | 7.1 | 446 | 6.0 | 659 | 6.1 | 626 | 6.2 | 551 |
| Education level |  |  |  |  |  |  |  |  |  |  |  |  |
| No education | 4.2 | 303 | 4.2 | 303 | 4.1 | 258 | 9.0 | 918 | 9.0 | 918 | 8.4 | 750 |
| Primary | 52.1 | 3795 | 52.1 | 3795 | 53.1 | 3348 | 59.2 | 6033 | 59.1 | 6033 | 59.7 | 5343 |
| Secondary | 37.0 | 2697 | 37.0 | 2697 | 36.9 | 2326 | 27.5 | 2809 | 27.5 | 2809 | 27.7 | 2481 |
| More than secondary | 6.7 | 486 | 6.7 | 486 | 5.9 | 373 | 4.3 | 439 | 4.3 | 439 | 4.1 | 371 |
| Missing |  |  | 0 | 2 | 0 | 1 |  |  | 0 | 4 | 0 | 4 |
| Ethnicity |  |  |  |  |  |  |  |  |  |  |  |  |
| Chewa | 27.6 | 2488 | 34.2 | 2488 | 35 | 2204 | 29.9 | 3287 | 32.2 | 3287 | 32.6 | 2917 |
| Lomwe | 15.6 | 1405 | 19.3 | 1405 | 18.5 | 1168 | 19.3 | 2127 | 20.8 | 2127 | 20.5 | 1837 |
| Ngoni | 9.8 | 884 | 12.1 | 884 | 12 | 757 | 11.5 | 1264 | 12.4 | 1264 | 12.6 | 1130 |
| Nkhonde | 0.8 | 75 | 1.0 | 75 | 1.1 | 68 | 0.9 | 103 | 1.0 | 103 | 1.1 | 95 |
| Sena | 3.3 | 301 | 4.1 | 301 | 4.2 | 265 | 3.8 | 416 | 4.1 | 416 | 4.0 | 360 |
| Tonga | 1.5 | 133 | 1.8 | 133 | 1.7 | 108 | 1.9 | 211 | 2.1 | 211 | 2.1 | 186 |
| Tumbuka | 9.2 | 829 | 11.4 | 829 | 12.0 | 756 | 9.8 | 1080 | 10.6 | 1080 | 11.0 | 986 |
| Yao | 8.5 | 772 | 10.6 | 772 | 10.1 | 638 | 10.4 | 1150 | 11.3 | 1150 | 10.5 | 940 |
| Other | 4.3 | 386 | 5.3 | 386 | 5.3 | 333 | 5.0 | 545 | 5.3 | 545 | 5.4 | 482 |
| Missing | 19.5 | 1757 | 0.1 | 10 | 0.1 | 9 | 7.5 | 823 | 0.2 | 20 | 0.2 | 16 |
| Marital status |  |  |  |  |  |  |  |  |  |  |  |  |
| Never married | 41.1 | 2990 | 41.1 | 2990 | 41.2 | 2596 | 21.7 | 2209 | 21.7 | 2209 | 21.9 | 1960 |
| Married or living together | 54.3 | 3953 | 54.3 | 3953 | 54.1 | 3412 | 64.5 | 6572 | 64.4 | 6572 | 63.8 | 5713 |
| Divorced or separated | 4.1 | 300 | 4.1 | 300 | 4.2 | 262 | 10.9 | 1112 | 10.9 | 1112 | 11.1 | 996 |
| Widowed | 0.4 | 31 | 0.4 | 31 | 0.5 | 29 | 2.9 | 298 | 2.9 | 298 | 3.0 | 269 |
| Missing |  |  | 0.1 | 9 | 0.1 | 7 |  |  | 0.1 | 12 | 0.1 | 11 |
| Circumcised |  |  |  |  |  |  |  |  |  |  |  |  |
| No | 87.2 | 6047 | 83.0 | 6047 | 83.1 | 5238 |  |  |  |  |  |  |
| Yes | 12.8 | 884 | 12.1 | 884 | 12.0 | 758 |  |  |  |  |  |  |
| Missing |  |  | 4.8 | 352 | 4.9 | 310 |  |  |  |  |  |  |
| Pregnant |  |  |  |  |  |  |  |  |  |  |  |  |
| No |  |  |  |  |  |  | 93.4 | 9397 | 92.1 | 9397 | 92.3 | 8263 |
| Yes |  |  |  |  |  |  | 6.6 | 669 | 6.6 | 669 | 6.3 | 562 |
| Missing |  |  |  |  |  |  |  |  | 1.3 | 137 | 1.4 | 124 |
| Ever tested for HIV |  |  |  |  |  |  |  |  |  |  |  |  |
| No | 33.2 | 2416 | 33.2 | 2416 | 33.6 | 2120 | 13.7 | 1401 | 13.7 | 1401 | 14.0 | 1254 |
| Yes | 66.8 | 4861 | 66.7 | 4861 | 66.3 | 4182 | 86.3 | 8794 | 86.2 | 8794 | 85.9 | 7687 |
| Missing |  |  | 0.1 | 6 | 0.1 | 4 |  |  | 0.1 | 8 | 0.1 | 8 |
| Ever had sex |  |  |  |  |  |  |  |  |  |  |  |  |
| No | 14.0 | 996 | 13.7 | 996 | 13.8 | 869 | 10.5 | 1067 | 10.5 | 1067 | 10.5 | 944 |
| Yes | 86.0 | 6093 | 83.7 | 6093 | 83.8 | 5285 | 89.5 | 9119 | 89.4 | 9119 | 89.3 | 7988 |
| Missing |  |  | 2.7 | 194 | 2.4 | 152 |  |  | 0.2 | 17 | 0.2 | 17 |

**Supplementary Table 2d. Sample characteristics for Zambia**

|  | **Males** | | | | | | **Females** | | | | | |
| --- | --- | --- | --- | --- | --- | --- | --- | --- | --- | --- | --- | --- |
|  | **All eligible household members** | | **Interview participants** | | **Blood test participants** | | **All eligible household members** | | **Interview participants** | | **Blood test participants** | |
| **Variable** | **%** | **N** | **%** | **N** | **%** | **N** | **%** | **N** | **%** | **N** | **%** | **N** |
| Geographic area (Province) |  |  |  |  |  |  |  |  |  |  |  |  |
| Central | 8.2 | 851 | 8.6 | 712 | 8.6 | 630 | 8.1 | 985 | 8.2 | 909 | 8.1 | 812 |
| Copperbelt | 17.0 | 1759 | 16.9 | 1407 | 16.9 | 1245 | 16.7 | 2037 | 16.9 | 1869 | 17.1 | 1715 |
| Eastern | 9.3 | 965 | 9.6 | 795 | 10.0 | 736 | 9.3 | 1128 | 9.4 | 1040 | 9.6 | 965 |
| Luapula | 4.8 | 500 | 5.2 | 430 | 5.2 | 386 | 5.1 | 623 | 5.3 | 583 | 5.2 | 521 |
| Lusaka | 19.0 | 1963 | 15.9 | 1320 | 15.4 | 1134 | 19.3 | 2354 | 18.6 | 2057 | 18.7 | 1869 |
| Muchinga | 8.8 | 910 | 9.3 | 769 | 9.3 | 686 | 8.8 | 1072 | 9.0 | 990 | 8.9 | 892 |
| Northern | 7.7 | 800 | 8.4 | 694 | 8.1 | 593 | 7.9 | 968 | 7.9 | 871 | 7.4 | 745 |
| North-Western | 9.5 | 981 | 9.2 | 766 | 9.2 | 679 | 9.6 | 1166 | 9.3 | 1028 | 9.3 | 935 |
| Southern | 10.9 | 1134 | 12.1 | 1003 | 12.4 | 911 | 10.3 | 1259 | 10.6 | 1167 | 10.7 | 1071 |
| Western | 4.8 | 495 | 4.9 | 409 | 4.9 | 358 | 4.9 | 600 | 4.9 | 538 | 4.8 | 485 |
| Urbanicity |  |  |  |  |  |  |  |  |  |  |  |  |
| Urban | 45.4 | 4705 | 41.9 | 3477 | 41.4 | 3047 | 46.4 | 5660 | 45.9 | 5071 | 46.3 | 4636 |
| Rural | 54.6 | 5653 | 58.1 | 4828 | 58.6 | 4311 | 53.6 | 6532 | 54.1 | 5981 | 53.7 | 5374 |
| Wealth quintile |  |  |  |  |  |  |  |  |  |  |  |  |
| Lowest | 14.7 | 1520 | 15.4 | 1282 | 15.3 | 1125 | 15.7 | 1919 | 15.9 | 1759 | 15.5 | 1547 |
| Second | 17.8 | 1840 | 19.1 | 1585 | 19.3 | 1418 | 17.4 | 2119 | 17.6 | 1942 | 17.4 | 1739 |
| Middle | 19.8 | 2056 | 20.7 | 1722 | 21.3 | 1567 | 19.1 | 2325 | 19.4 | 2145 | 19.6 | 1966 |
| Fourth | 22.3 | 2307 | 20.9 | 1738 | 20.8 | 1533 | 22.2 | 2707 | 22.3 | 2463 | 22.5 | 2255 |
| Highest | 24.9 | 2577 | 23.3 | 1938 | 22.8 | 1678 | 25.2 | 3070 | 24.4 | 2700 | 24.6 | 2461 |
| Missing | 0.6 | 58 | 0.5 | 40 | 0.5 | 37 | 0.4 | 52 | 0.4 | 43 | 0.4 | 42 |
| Age group |  |  |  |  |  |  |  |  |  |  |  |  |
| 15-19 | 24.3 | 2512 | 24.2 | 2006 | 24.6 | 1811 | 22.7 | 2766 | 21.1 | 2331 | 21.2 | 2120 |
| 20-24 | 17.6 | 1825 | 18.0 | 1496 | 18.3 | 1344 | 20.0 | 2439 | 20.4 | 2256 | 20.4 | 2045 |
| 25-29 | 14.3 | 1484 | 14.6 | 1209 | 14.3 | 1053 | 16.1 | 1962 | 16.3 | 1804 | 16.2 | 1619 |
| 30-34 | 14.3 | 1478 | 13.9 | 1157 | 13.6 | 1003 | 14.3 | 1742 | 14.6 | 1614 | 14.6 | 1458 |
| 35-39 | 11.7 | 1215 | 11.5 | 956 | 11.4 | 836 | 11.4 | 1385 | 11.8 | 1299 | 11.6 | 1160 |
| 40-45 | 10.5 | 1085 | 10.2 | 851 | 10.2 | 751 | 9.5 | 1159 | 9.7 | 1071 | 9.9 | 989 |
| 45-49 | 7.3 | 759 | 7.6 | 630 | 7.6 | 560 | 6.1 | 739 | 6.1 | 677 | 6.2 | 619 |
| Education level |  |  |  |  |  |  |  |  |  |  |  |  |
| No education | 3.1 | 256 | 3.1 | 256 | 3.0 | 220 | 6.5 | 713 | 6.5 | 713 | 6.1 | 606 |
| Primary | 37.2 | 3086 | 37.2 | 3086 | 37.3 | 2742 | 45.3 | 4999 | 45.2 | 4999 | 45.4 | 4546 |
| Secondary | 50.2 | 4162 | 50.1 | 4162 | 50.8 | 3737 | 41.3 | 4563 | 41.3 | 4563 | 41.8 | 4181 |
| More than secondary | 9.5 | 792 | 9.5 | 792 | 8.8 | 651 | 7.0 | 768 | 6.9 | 768 | 6.7 | 674 |
| Missing |  |  | 0.1 | 9 | 0.1 | 8 |  |  | 0.1 | 9 | 0 | 3 |
| Ethnicity |  |  |  |  |  |  |  |  |  |  |  |  |
| Bemba | 23.4 | 2428 | 29.2 | 2428 | 29.0 | 2137 | 26.8 | 3270 | 29.6 | 3270 | 29.2 | 2918 |
| Tonga | 11.4 | 1180 | 14.2 | 1180 | 14.4 | 1061 | 12.0 | 1466 | 13.3 | 1466 | 13.3 | 1330 |
| Kaonde | 2.7 | 276 | 3.3 | 276 | 3.2 | 232 | 3.7 | 451 | 4.1 | 451 | 4.0 | 402 |
| Lozi | 5.6 | 582 | 7.0 | 582 | 6.9 | 508 | 5.8 | 707 | 6.4 | 707 | 6.5 | 651 |
| Lunda | 3.6 | 377 | 4.5 | 377 | 4.6 | 340 | 3.4 | 417 | 3.8 | 417 | 3.8 | 382 |
| Luvale | 2.6 | 265 | 3.2 | 265 | 3.2 | 232 | 3.4 | 416 | 3.8 | 416 | 3.7 | 371 |
| Mambwe | 2.2 | 227 | 2.7 | 227 | 2.6 | 194 | 2.9 | 356 | 3.2 | 356 | 3.1 | 312 |
| Ngoni | 3.9 | 408 | 4.9 | 408 | 5.0 | 369 | 3.8 | 465 | 4.2 | 465 | 4.4 | 436 |
| Nyanja | 6.1 | 631 | 7.6 | 631 | 7.6 | 561 | 5.8 | 713 | 6.5 | 713 | 6.4 | 644 |
| Tumbuka | 3.9 | 403 | 4.9 | 403 | 5.1 | 377 | 4.4 | 531 | 4.8 | 531 | 4.9 | 495 |
| Other | 14.6 | 1516 | 18.3 | 1516 | 18.2 | 1337 | 18.4 | 2247 | 20.3 | 2247 | 20.6 | 2060 |
| Missing | 19.9 | 2065 | 0.1 | 12 | 0.1 | 10 | 9.5 | 1153 | 0.1 | 13 | 0.1 | 9 |
| Marital status |  |  |  |  |  |  |  |  |  |  |  |  |
| Never married | 45.6 | 3750 | 45.2 | 3750 | 45.5 | 3345 | 29.7 | 3260 | 29.5 | 3260 | 29.6 | 2959 |
| Married or living together | 49.9 | 4106 | 49.4 | 4106 | 49.2 | 3620 | 58.0 | 6362 | 57.6 | 6362 | 57.4 | 5742 |
| Divorced or separated | 4.1 | 334 | 4.0 | 334 | 4.0 | 293 | 8.9 | 975 | 8.8 | 975 | 8.9 | 895 |
| Widowed | 0.5 | 42 | 0.5 | 42 | 0.5 | 40 | 3.3 | 367 | 3.3 | 367 | 3.4 | 336 |
| Missing |  |  | 0.9 | 73 | 0.8 | 60 |  |  | 0.8 | 88 | 0.8 | 78 |
| Circumcised |  |  |  |  |  |  |  |  |  |  |  |  |
| No | 77.8 | 6352 | 76.5 | 6352 | 76.6 | 5635 |  |  |  |  |  |  |
| Yes | 22.2 | 1817 | 21.9 | 1817 | 21.8 | 1602 |  |  |  |  |  |  |
| Missing |  |  | 1.6 | 136 | 1.6 | 121 |  |  |  |  |  |  |
| Pregnant |  |  |  |  |  |  |  |  |  |  |  |  |
| No |  |  |  |  |  |  | 91.9 | 10012 | 90.6 | 10012 | 90.6 | 9067 |
| Yes |  |  |  |  |  |  | 8.1 | 877 | 7.9 | 877 | 7.9 | 789 |
| Missing |  |  |  |  |  |  |  |  | 1.5 | 163 | 1.5 | 154 |
| Ever tested for HIV |  |  |  |  |  |  |  |  |  |  |  |  |
| No | 34.2 | 2833 | 34.1 | 2833 | 34.4 | 2532 | 18.1 | 1996 | 18.1 | 1996 | 18.1 | 1815 |
| Yes | 65.8 | 5456 | 65.7 | 5456 | 65.4 | 4811 | 81.9 | 9041 | 81.8 | 9041 | 81.8 | 8185 |
| Missing |  |  | 0.2 | 16 | 0.2 | 15 |  |  | 0.1 | 15 | 0.1 | 10 |
| Ever had sex |  |  |  |  |  |  |  |  |  |  |  |  |
| No | 17.0 | 1336 | 16.1 | 1336 | 15.9 | 1170 | 13.3 | 1462 | 13.2 | 1462 | 13.0 | 1300 |
| Yes | 83.0 | 6538 | 78.7 | 6538 | 79.2 | 5828 | 86.7 | 9536 | 86.3 | 9536 | 86.5 | 8663 |
| Missing |  |  | 5.2 | 431 | 4.9 | 360 |  |  | 0.5 | 54 | 0.5 | 47 |

**Supplementary Table 2e. Sample characteristics for Zimbabwe**

|  | **Males** | | | | | | **Females** | | | | | |
| --- | --- | --- | --- | --- | --- | --- | --- | --- | --- | --- | --- | --- |
|  | **All eligible household members** | | **Interview participants** | | **Blood test participants** | | **All eligible household members** | | **Interview participants** | | **Blood test participants** | |
| **Variable** | **%** | **N** | **%** | **N** | **%** | **N** | **%** | **N** | **%** | **N** | **%** | **N** |
| Geographic area (Province) |  |  |  |  |  |  |  |  |  |  |  |  |
| Bulawayo | 8.5 | 823 | 7.9 | 632 | 8.0 | 576 | 10.5 | 1235 | 10.4 | 1158 | 10.6 | 1079 |
| Manicaland | 10.3 | 997 | 10.8 | 870 | 11.2 | 813 | 10.3 | 1212 | 10.5 | 1164 | 10.5 | 1074 |
| Mashonaland Central | 11.7 | 1137 | 11.9 | 952 | 11.5 | 834 | 9.8 | 1160 | 9.6 | 1065 | 9.3 | 946 |
| Mashonaland East | 10.1 | 983 | 10.8 | 864 | 10.8 | 782 | 8.4 | 994 | 8.6 | 950 | 8.6 | 883 |
| Mashonaland West | 13.2 | 1280 | 13.9 | 1116 | 14.1 | 1020 | 11.1 | 1306 | 11.1 | 1233 | 11.1 | 1132 |
| Matebeland North | 8.8 | 852 | 9.1 | 728 | 9.1 | 660 | 9.3 | 1099 | 9.3 | 1036 | 9.4 | 959 |
| Matebeland South | 7.0 | 675 | 7.0 | 559 | 7.0 | 510 | 7.3 | 864 | 7.4 | 820 | 7.4 | 761 |
| Midlands | 9.5 | 925 | 9.2 | 737 | 9.0 | 654 | 10.4 | 1224 | 10.4 | 1151 | 10.2 | 1047 |
| Masvingo | 9.4 | 916 | 10.0 | 803 | 9.8 | 712 | 10.9 | 1286 | 10.9 | 1207 | 11.1 | 1136 |
| Harare | 11.5 | 1114 | 9.5 | 759 | 9.4 | 680 | 12.1 | 1426 | 11.8 | 1306 | 11.8 | 1204 |
| Urbanicity |  |  |  |  |  |  |  |  |  |  |  |  |
| Urban | 30.5 | 2955 | 26.9 | 2154 | 26.5 | 1917 | 35.0 | 4129 | 34.5 | 3829 | 34.2 | 3500 |
| Rural | 69.5 | 6747 | 73.1 | 5866 | 73.5 | 5324 | 65.0 | 7677 | 65.5 | 7261 | 65.8 | 6721 |
| Wealth quintile |  |  |  |  |  |  |  |  |  |  |  |  |
| Lowest | 22.6 | 2189 | 23.5 | 1883 | 23.6 | 1711 | 22.3 | 2630 | 22.4 | 2488 | 22.4 | 2290 |
| Second | 20.1 | 1946 | 21.6 | 1734 | 21.9 | 1588 | 19.4 | 2285 | 19.4 | 2154 | 19.6 | 2008 |
| Middle | 20.7 | 2008 | 21.4 | 1713 | 21.5 | 1560 | 17.2 | 2034 | 17.4 | 1929 | 17.6 | 1799 |
| Fourth | 18.0 | 1742 | 16.5 | 1326 | 16.4 | 1187 | 18.5 | 2183 | 18.4 | 2046 | 18.4 | 1877 |
| Highest | 18.7 | 1817 | 17.0 | 1364 | 16.5 | 1195 | 22.6 | 2673 | 22.3 | 2473 | 22.0 | 2247 |
| Missing | 0 | 0 | 0 | 0 | 0 | 0 | 0 | 1 | 0 | 0 | 0 | 0 |
| Age group |  |  |  |  |  |  |  |  |  |  |  |  |
| 15-19 | 26.1 | 2537 | 26.3 | 2112 | 26.9 | 1950 | 21.1 | 2486 | 20.5 | 2275 | 20.7 | 2114 |
| 20-24 | 16.7 | 1616 | 17.1 | 1373 | 16.8 | 1220 | 17.8 | 2104 | 17.9 | 1982 | 17.8 | 1817 |
| 25-29 | 14.0 | 1356 | 13.4 | 1077 | 13.5 | 979 | 15.5 | 1833 | 15.6 | 1728 | 15.4 | 1573 |
| 30-34 | 13.8 | 1340 | 13.4 | 1076 | 13.0 | 942 | 15.2 | 1790 | 15.3 | 1697 | 15.4 | 1579 |
| 35-39 | 11.9 | 1152 | 11.8 | 943 | 11.6 | 843 | 12.9 | 1524 | 13.0 | 1444 | 13.0 | 1326 |
| 40-45 | 10.2 | 992 | 10.4 | 831 | 10.4 | 754 | 10.3 | 1212 | 10.3 | 1145 | 10.4 | 1063 |
| 45-49 | 7.3 | 709 | 7.6 | 608 | 7.6 | 553 | 7.3 | 857 | 7.4 | 819 | 7.3 | 749 |
| Education level |  |  |  |  |  |  |  |  |  |  |  |  |
| No education | 0.7 | 58 | 0.7 | 58 | 0.7 | 52 | 1.5 | 166 | 1.5 | 166 | 1.5 | 150 |
| Primary | 24.2 | 1940 | 24.2 | 1940 | 24.6 | 1780 | 26.2 | 2905 | 26.2 | 2905 | 26.3 | 2691 |
| Secondary | 66.4 | 5326 | 66.4 | 5326 | 66.6 | 4826 | 66.4 | 7360 | 66.4 | 7360 | 66.6 | 6806 |
| More than secondary | 8.6 | 693 | 8.6 | 693 | 8.0 | 580 | 5.9 | 655 | 5.9 | 655 | 5.6 | 570 |
| Missing |  |  | 0 | 3 | 0 | 3 |  |  | 0 | 4 | 0 | 4 |
| Marital status |  |  |  |  |  |  |  |  |  |  |  |  |
| Never married | 46.2 | 3701 | 46.1 | 3701 | 46.2 | 3348 | 25.3 | 2804 | 25.3 | 2804 | 25.5 | 2604 |
| Married or living together | 48.6 | 3891 | 48.5 | 3891 | 48.5 | 3515 | 61.1 | 6764 | 61.0 | 6764 | 60.8 | 6215 |
| Divorced or separated | 4.4 | 349 | 4.4 | 349 | 4.2 | 307 | 8.5 | 946 | 8.5 | 946 | 8.5 | 871 |
| Widowed | 0.8 | 66 | 0.8 | 66 | 0.8 | 61 | 5.1 | 560 | 5.0 | 560 | 5.1 | 517 |
| Missing |  |  | 0.2 | 13 | 0.1 | 10 |  |  | 0.1 | 16 | 0.1 | 14 |
| Circumcised |  |  |  |  |  |  |  |  |  |  |  |  |
| No | 86.3 | 6694 | 83.5 | 6694 | 83.4 | 6039 |  |  |  |  |  |  |
| Yes | 13.7 | 1059 | 13.2 | 1059 | 13.4 | 967 |  |  |  |  |  |  |
| Missing |  |  | 3.3 | 267 | 3.2 | 235 |  |  |  |  |  |  |
| Pregnant |  |  |  |  |  |  |  |  |  |  |  |  |
| No |  |  |  |  |  |  | 93.8 | 10207 | 92.0 | 10207 | 92.2 | 9419 |
| Yes |  |  |  |  |  |  | 6.2 | 676 | 6.1 | 676 | 5.9 | 608 |
| Missing |  |  |  |  |  |  |  |  | 1.9 | 207 | 1.9 | 194 |
| Ever tested for HIV |  |  |  |  |  |  |  |  |  |  |  |  |
| No | 34.1 | 2732 | 34.1 | 2732 | 33.7 | 2440 | 16.2 | 1796 | 16.2 | 1796 | 15.5 | 1587 |
| Yes | 65.9 | 5273 | 65.7 | 5273 | 66.1 | 4789 | 83.8 | 9290 | 83.8 | 9290 | 84.5 | 8633 |
| Missing |  |  | 0.2 | 15 | 0.2 | 12 |  |  | 0 | 4 | 0 | 1 |
| Ever had sex |  |  |  |  |  |  |  |  |  |  |  |  |
| No | 23.7 | 1861 | 23.2 | 1861 | 23.3 | 1687 | 16.3 | 1801 | 16.2 | 1801 | 16.2 | 1658 |
| Yes | 76.3 | 6005 | 74.9 | 6005 | 74.9 | 5427 | 83.7 | 9265 | 83.5 | 9265 | 83.6 | 8540 |
| Missing |  |  | 1.9 | 154 | 1.8 | 127 |  |  | 0.2 | 24 | 0.2 | 23 |

**Supplementary Table 2f. Sample characteristics for Lesotho**

|  | **Males** | | | | | | **Females** | | | | | |
| --- | --- | --- | --- | --- | --- | --- | --- | --- | --- | --- | --- | --- |
|  | **All eligible household members** | | **Interview participants** | | **Blood test participants** | | **All eligible household members** | | **Interview participants** | | **Blood test participants** | |
| **Variable** | **%** | **N** | **%** | **N** | **%** | **N** | **%** | **N** | **%** | **N** | **%** | **N** |
| Geographic area (District) |  |  |  |  |  |  |  |  |  |  |  |  |
| Maseru | 6.9 | 379 | 6.6 | 314 | 6.1 | 258 | 6.3 | 433 | 6.1 | 398 | 6.0 | 362 |
| Mafeteng | 16.8 | 922 | 16.7 | 795 | 17.0 | 715 | 16.6 | 1144 | 16.8 | 1100 | 17.0 | 1020 |
| Mohale's Hoek | 13.5 | 741 | 13.1 | 621 | 12.8 | 539 | 13.2 | 904 | 13.2 | 864 | 12.9 | 772 |
| Leribe | 27.8 | 1519 | 27.3 | 1296 | 26.1 | 1096 | 26.7 | 1832 | 26.0 | 1702 | 25.0 | 1495 |
| Berea | 9.2 | 505 | 9.8 | 464 | 10.4 | 436 | 9.3 | 640 | 9.5 | 624 | 9.9 | 595 |
| Quthing | 6.6 | 363 | 6.8 | 321 | 6.9 | 288 | 7.2 | 494 | 7.3 | 475 | 7.3 | 440 |
| Butha-Buthe | 4.6 | 252 | 4.7 | 225 | 4.7 | 198 | 5.1 | 351 | 5.2 | 338 | 5.4 | 323 |
| Mokhotlong | 3.3 | 183 | 3.5 | 167 | 3.5 | 146 | 3.8 | 263 | 3.9 | 257 | 3.9 | 236 |
| Qacha's Nek | 5.5 | 303 | 5.4 | 257 | 5.8 | 243 | 6.0 | 411 | 5.9 | 384 | 6.1 | 363 |
| Thaba-Tseka | 5.6 | 306 | 6.1 | 292 | 6.7 | 280 | 5.8 | 399 | 6.0 | 394 | 6.4 | 384 |
| Urbanicity |  |  |  |  |  |  |  |  |  |  |  |  |
| Urban/peri-urban | 45.9 | 2512 | 45.6 | 2166 | 43.7 | 1837 | 49.4 | 3396 | 49.1 | 3207 | 47.7 | 2855 |
| Rural | 54.1 | 2961 | 54.4 | 2586 | 56.3 | 2362 | 50.6 | 3475 | 50.9 | 3329 | 52.3 | 3135 |
| Wealth quintile |  |  |  |  |  |  |  |  |  |  |  |  |
| Lowest | 18.7 | 1025 | 18.9 | 900 | 19.7 | 828 | 18.6 | 1281 | 18.6 | 1216 | 19.4 | 1162 |
| Second | 20.2 | 1104 | 20.2 | 960 | 21.3 | 894 | 18.2 | 1251 | 18.3 | 1199 | 18.9 | 1131 |
| Middle | 19.9 | 1088 | 20.2 | 962 | 20.4 | 855 | 19.7 | 1352 | 19.9 | 1299 | 20.2 | 1209 |
| Fourth | 20.6 | 1129 | 20.8 | 987 | 20.6 | 864 | 20.6 | 1417 | 20.7 | 1352 | 20.3 | 1215 |
| Highest | 20.3 | 1112 | 19.6 | 931 | 17.8 | 746 | 22.6 | 1551 | 22.2 | 1454 | 21.0 | 1257 |
| Missing | 0.3 | 15 | 0.3 | 12 | 0.3 | 12 | 0.3 | 19 | 0.2 | 16 | 0.3 | 16 |
| Age group |  |  |  |  |  |  |  |  |  |  |  |  |
| 15-19 | 21.1 | 1157 | 21.1 | 1001 | 21.9 | 921 | 19.7 | 1351 | 19.1 | 1247 | 19.3 | 1156 |
| 20-24 | 18.3 | 1002 | 18.4 | 875 | 18.3 | 769 | 19.7 | 1357 | 19.8 | 1297 | 20.1 | 1202 |
| 25-29 | 17.6 | 961 | 17.4 | 827 | 16.8 | 707 | 17.6 | 1211 | 17.7 | 1159 | 17.6 | 1054 |
| 30-34 | 14.9 | 818 | 14.7 | 697 | 14.6 | 611 | 14.6 | 1003 | 14.8 | 965 | 14.3 | 857 |
| 35-39 | 12.2 | 665 | 12.1 | 573 | 11.8 | 497 | 11.7 | 802 | 11.8 | 769 | 11.7 | 703 |
| 40-45 | 8.9 | 486 | 9.0 | 427 | 9.0 | 379 | 9.5 | 654 | 9.6 | 625 | 9.6 | 574 |
| 45-49 | 7.0 | 384 | 7.4 | 352 | 7.5 | 315 | 7.2 | 493 | 7.3 | 474 | 7.4 | 444 |
| Education level |  |  |  |  |  |  |  |  |  |  |  |  |
| No education | 7.8 | 371 | 7.8 | 371 | 8.1 | 342 | 1.4 | 89 | 1.4 | 89 | 1.3 | 80 |
| Primary | 40.2 | 1906 | 40.1 | 1906 | 41.7 | 1752 | 36.1 | 2358 | 36.1 | 2358 | 37.1 | 2224 |
| Secondary | 42.2 | 2004 | 42.2 | 2004 | 41.4 | 1740 | 51.8 | 3387 | 51.8 | 3387 | 52.1 | 3122 |
| More than secondary | 9.8 | 466 | 9.8 | 466 | 8.6 | 360 | 10.7 | 701 | 10.7 | 701 | 9.4 | 563 |
| Missing |  |  | 0.1 | 5 | 0.1 | 5 |  |  | 0 | 1 | 0 | 1 |
| Marital status |  |  |  |  |  |  |  |  |  |  |  |  |
| Never married | 52.6 | 2492 | 52.4 | 2492 | 52.6 | 2207 | 33.9 | 2211 | 33.8 | 2211 | 33.1 | 1985 |
| Married or living together | 39.5 | 1872 | 39.4 | 1872 | 39.2 | 1644 | 52.6 | 3435 | 52.6 | 3435 | 53.0 | 3176 |
| Divorced or separated | 5.8 | 274 | 5.8 | 274 | 5.8 | 245 | 6.4 | 419 | 6.4 | 419 | 6.5 | 389 |
| Widowed | 2.1 | 98 | 2.1 | 98 | 2.1 | 89 | 7.1 | 465 | 7.1 | 465 | 7.3 | 435 |
| Missing |  |  | 0.3 | 16 | 0.3 | 14 |  |  | 0.1 | 6 | 0.1 | 5 |
| Circumcised |  |  |  |  |  |  |  |  |  |  |  |  |
| No | 62.9 | 2942 | 61.9 | 2942 | 63.3 | 2657 |  |  |  |  |  |  |
| Yes | 37.1 | 1736 | 36.5 | 1736 | 35.2 | 1478 |  |  |  |  |  |  |
| Missing |  |  | 1.6 | 74 | 1.5 | 64 |  |  |  |  |  |  |
| Pregnant |  |  |  |  |  |  |  |  |  |  |  |  |
| No |  |  |  |  |  |  | 95.5 | 6218 | 95.1 | 6218 | 95.1 | 5695 |
| Yes |  |  |  |  |  |  | 4.5 | 291 | 4.5 | 291 | 4.6 | 273 |
| Missing |  |  |  |  |  |  |  |  | 0.4 | 27 | 0.4 | 22 |
| Ever tested for HIV |  |  |  |  |  |  |  |  |  |  |  |  |
| No | 17.7 | 840 | 17.7 | 840 | 17.6 | 739 | 8.3 | 543 | 8.3 | 543 | 7.8 | 466 |
| Yes | 82.3 | 3911 | 82.3 | 3911 | 82.4 | 3459 | 91.7 | 5991 | 91.7 | 5991 | 92.2 | 5522 |
| Missing |  |  | 0 | 1 | 0 | 1 |  |  | 0 | 2 | 0 | 2 |
| Ever had sex |  |  |  |  |  |  |  |  |  |  |  |  |
| No | 14.7 | 698 | 14.7 | 698 | 15.1 | 636 | 13.2 | 866 | 13.2 | 866 | 13.1 | 783 |
| Yes | 85.3 | 4052 | 85.3 | 4052 | 84.8 | 3561 | 86.8 | 5670 | 86.8 | 5670 | 86.9 | 5207 |
| Missing |  |  | 0 | 2 | 0 | 2 |  |  | 0 | 0 | 0 | 0 |

**Supplementary Table 2g. Sample characteristics for Eswatini**

|  | **Males** | | | | | | **Females** | | | | | |
| --- | --- | --- | --- | --- | --- | --- | --- | --- | --- | --- | --- | --- |
|  | **All eligible household members** | | **Interview participants** | | **Blood test participants** | | **All eligible household members** | | **Interview participants** | | **Blood test participants** | |
| **Variable** | **%** | **N** | **%** | **N** | **%** | **N** | **%** | **N** | **%** | **N** | **%** | **N** |
| Geographic area (Region) |  |  |  |  |  |  |  |  |  |  |  |  |
| Hhohho | 31.1 | 1442 | 31.7 | 1266 | 31.7 | 1160 | 29.5 | 1630 | 30.0 | 1545 | 29.7 | 1451 |
| Lubombo | 20.2 | 935 | 20.3 | 811 | 21.1 | 772 | 19.5 | 1076 | 19.4 | 1000 | 19.9 | 973 |
| Manzini | 31.2 | 1447 | 31.1 | 1240 | 30.6 | 1117 | 32.8 | 1810 | 32.7 | 1689 | 32.6 | 1592 |
| Shiselweni | 17.6 | 815 | 16.8 | 671 | 16.6 | 606 | 18.3 | 1009 | 17.9 | 924 | 17.7 | 862 |
| Urbanicity |  |  |  |  |  |  |  |  |  |  |  |  |
| Urban | 23.5 | 1090 | 23.9 | 952 | 23.0 | 842 | 23.5 | 1301 | 23.2 | 1199 | 22.6 | 1100 |
| Rural | 76.5 | 3549 | 76.1 | 3036 | 77.0 | 2813 | 76.5 | 4224 | 76.8 | 3959 | 77.4 | 3778 |
| Wealth quintile |  |  |  |  |  |  |  |  |  |  |  |  |
| Lowest | 21.3 | 989 | 21.5 | 858 | 22.3 | 815 | 21.8 | 1202 | 21.8 | 1126 | 22.7 | 1105 |
| Second | 20.6 | 954 | 20.8 | 830 | 21.2 | 775 | 20.7 | 1144 | 21.0 | 1081 | 21.2 | 1035 |
| Middle | 23.6 | 1095 | 23.3 | 929 | 23.6 | 863 | 23.0 | 1271 | 22.9 | 1182 | 23.2 | 1131 |
| Fourth | 17.4 | 807 | 17.3 | 689 | 16.9 | 618 | 16.9 | 935 | 17.1 | 882 | 16.8 | 820 |
| Highest | 17.1 | 791 | 17.1 | 681 | 16.0 | 583 | 17.6 | 971 | 17.2 | 885 | 16.1 | 785 |
| Missing | 0.1 | 3 | 0 | 1 | 0 | 1 | 0 | 2 | 0 | 2 | 0 | 2 |
| Age group |  |  |  |  |  |  |  |  |  |  |  |  |
| 15-19 | 26.2 | 1216 | 26.9 | 1074 | 28.1 | 1028 | 20.8 | 1148 | 20.6 | 1064 | 21.1 | 1031 |
| 20-24 | 18.0 | 837 | 17.8 | 709 | 17.7 | 647 | 18.5 | 1021 | 18.4 | 950 | 18.3 | 895 |
| 25-29 | 16.3 | 754 | 16.1 | 642 | 15.6 | 572 | 16.7 | 921 | 16.7 | 861 | 16.6 | 811 |
| 30-34 | 14.0 | 649 | 13.6 | 541 | 13.0 | 474 | 15.7 | 866 | 15.7 | 811 | 15.3 | 744 |
| 35-39 | 11.6 | 536 | 11.4 | 453 | 11.2 | 410 | 11.7 | 645 | 11.7 | 602 | 11.6 | 568 |
| 40-45 | 7.8 | 364 | 8.1 | 323 | 8.0 | 292 | 8.7 | 479 | 8.7 | 450 | 8.8 | 430 |
| 45-49 | 6.1 | 283 | 6.2 | 246 | 6.3 | 232 | 8.1 | 445 | 8.1 | 420 | 8.2 | 399 |
| Education level |  |  |  |  |  |  |  |  |  |  |  |  |
| No education | 2.7 | 108 | 2.7 | 108 | 2.7 | 100 | 2.8 | 144 | 2.8 | 144 | 2.9 | 141 |
| Primary | 27.7 | 1103 | 27.7 | 1103 | 28.9 | 1057 | 24.3 | 1250 | 24.2 | 1250 | 25.2 | 1231 |
| Secondary | 28.0 | 1117 | 28.0 | 1117 | 28.8 | 1054 | 33.7 | 1734 | 33.6 | 1734 | 34.2 | 1667 |
| More than secondary | 41.6 | 1658 | 41.6 | 1658 | 39.5 | 1442 | 39.3 | 2022 | 39.2 | 2022 | 37.5 | 1831 |
| Missing |  |  | 0.1 | 2 | 0.1 | 2 |  |  | 0.2 | 8 | 0.2 | 8 |
| Marital status |  |  |  |  |  |  |  |  |  |  |  |  |
| Never married | 68.3 | 2706 | 67.9 | 2706 | 68.0 | 2485 | 53.4 | 2740 | 53.1 | 2740 | 53.0 | 2584 |
| Married or living together | 27.8 | 1100 | 27.6 | 1100 | 27.3 | 999 | 39.2 | 2011 | 39.0 | 2011 | 39.1 | 1909 |
| Divorced or separated | 3.5 | 137 | 3.4 | 137 | 3.5 | 128 | 3.8 | 196 | 3.8 | 196 | 3.7 | 181 |
| Widowed | 0.5 | 20 | 0.5 | 20 | 0.5 | 20 | 3.5 | 182 | 3.5 | 182 | 3.6 | 175 |
| Missing |  |  | 0.6 | 25 | 0.6 | 23 |  |  | 0.6 | 29 | 0.6 | 29 |
| Circumcised |  |  |  |  |  |  |  |  |  |  |  |  |
| No | 70.6 | 2773 | 69.5 | 2773 | 69.5 | 2542 |  |  |  |  |  |  |
| Yes | 29.4 | 1157 | 29.0 | 1157 | 29.1 | 1065 |  |  |  |  |  |  |
| Missing |  |  | 1.5 | 58 | 1.3 | 48 |  |  |  |  |  |  |
| Pregnant |  |  |  |  |  |  |  |  |  |  |  |  |
| No |  |  |  |  |  |  | 96.0 | 4896 | 94.9 | 4896 | 94.9 | 4627 |
| Yes |  |  |  |  |  |  | 4.0 | 202 | 3.9 | 202 | 3.9 | 191 |
| Missing |  |  |  |  |  |  |  |  | 1.2 | 60 | 1.2 | 60 |
| Ever tested for HIV |  |  |  |  |  |  |  |  |  |  |  |  |
| No | 18.3 | 728 | 18.3 | 728 | 17.9 | 653 | 8.0 | 412 | 8.0 | 412 | 8.1 | 394 |
| Yes | 81.7 | 3252 | 81.5 | 3252 | 82.0 | 2997 | 92.0 | 4737 | 91.8 | 4737 | 91.8 | 4477 |
| Missing |  |  | 0.2 | 8 | 0.1 | 5 |  |  | 0.2 | 9 | 0.1 | 7 |
| Ever had sex |  |  |  |  |  |  |  |  |  |  |  |  |
| No | 27.5 | 1081 | 27.1 | 1081 | 28.1 | 1027 | 17.0 | 873 | 16.9 | 873 | 17.3 | 842 |
| Yes | 72.5 | 2844 | 71.3 | 2844 | 70.3 | 2569 | 83.0 | 4266 | 82.7 | 4266 | 82.4 | 4020 |
| Missing |  |  | 1.6 | 63 | 1.6 | 59 |  |  | 0.4 | 19 | 0.3 | 16 |

**Supplementary Table 3. Interviewer-level participation rate (%) and HIV test prevalence (%)**

|  | **Number of interviewers** | | **Number of attempts^a^** | | **Participation rate^a^ (%)** | | **HIV prevalence^a^ (%)** | | **Pearson’s r** |
| --- | --- | --- | --- | --- | --- | --- | --- | --- | --- |
| **Country** | **Total** | **>10 attempts** | **Mean (SD)** | **Range** | **Mean (SD)** | **Range** | **Mean (SD)** | **Range** | **correlation^b^** |
| Tanzania | 198 | 181 | 150.0 (39.5) | 17-246 | 94.0 (3.4) | 80.4-100.0 | 5.5 (2.6) | 0.0-14.4 | .087 |
| Uganda | 237 | 211 | 123.5 (65.5) | 12-355 | 98.0 (2.1) | 92.1-100.0 | 5.2 (3.7) | 0.0-18.8 | -.060 |
| Malawi | 185 | 170 | 106.8 (42.8) | 11-219 | 83.4 (8.0) | 27.3-100.0 | 12.2 (5.2) | 0.0-28.6 | -.031 |
| Zambia | 341 | 290 | 69.1 (42.9) | 13-207 | 85.2 (10.6) | 25.0-100.0 | 11.3 (6.5) | 0.0-42.9 | .246 |
| Zimbabwe | 184 | 150 | 131.4 (52.1) | 11-284 | 87.7 (6.2) | 48.5-100.0 | 15.5 (5.0) | 4.7-33.3 | .062 |
| Lesotho | 218 | 152 | 74.0 (36.7) | 11-194 | 87.4 (12.0) | 25.0-100.0 | 25.7 (7.2) | 0.0-44.4 | -.083 |
| Eswatini | 103 | 89 | 107.6 (29.9) | 11-170 | 88.3 (5.7) | 61.5-100.0 | 28.4 (6.0) | 17.7-44.8 | .003 |
| ^a^ Interviewer-level Participation rate and HIV prevalence were calculated only among interviewers with >10 attempts to elicit blood test consent.  ^b^ Correlation between participation rate and HIV prevalence were estimated using Pearson’s r correlation coefficient, weighted by number of attempts per interviewer. | | | | | | | | | |


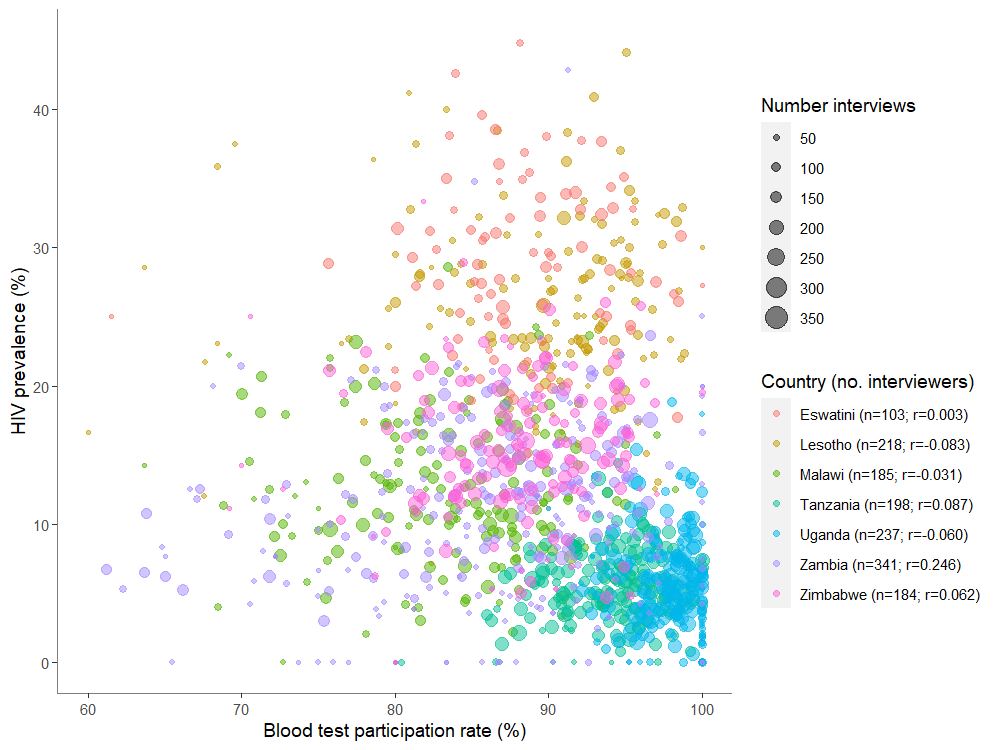


**Supplementary Figure 1. Consent rates and HIV prevalence by interviewer.** Each circle represents one interviewer and each color indicates a different country (see key).
Circle size indicates number of interviews attempted, including respondents and non-respondents. The key shows in parentheses the number of interviewers by survey and Pearson r correlations between interviewer-level test participation rate (%) and HIV prevalence (%), where positive r indicates that interviewers who are more successful at obtaining consent (as a percent of total number of blood test consent attempts) had higher HIV prevalence among those they consented.


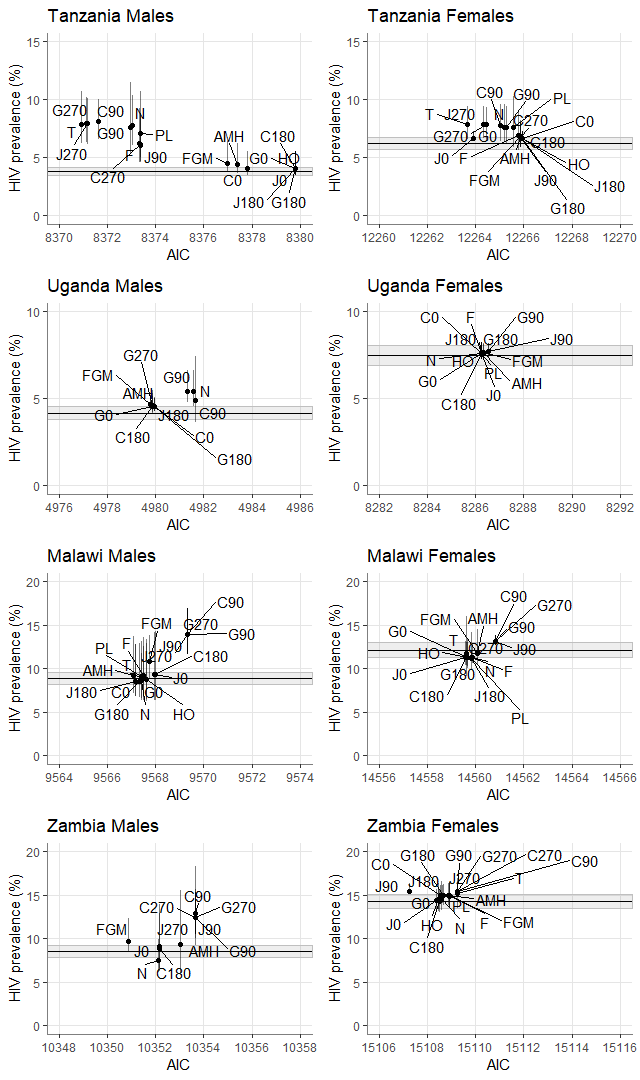


**Supplementary Figure 2a. Selection model results by copula.** Plots of selection model results by country and sex for each bivariate copula, AIC (x-axis) vs. HIV prevalence estimate and 95% confidence interval (y-axis). Points further left indicate better model fit (lower AIC). Note that axis ranges differ across surveys. Horizontal black line and grey rectangle denote IPW estimate and 95% confidence interval. Point labels denote copula used and are color-coded with red indicating higher HIV prevalence estimates and blue indicating lower estimates, relative to other copulae in the country: “N” = Normal, “F” = Frank, “T” = Student-t, “FGM” = Farlie-Gumbel-Morgenstern, “AMH” = Ali-Mikhail-Haq, “PL” = Placket, “HO” = Hougaard, “J” = Joe, “C” = Clayton, “G” = Gumbel. For Joe, Clayton and Gumbel copulae, numbers indicate rotation, either 0, 90, 180 or 270 degrees. Boxed label is the copula chosen for the selection model estimate.


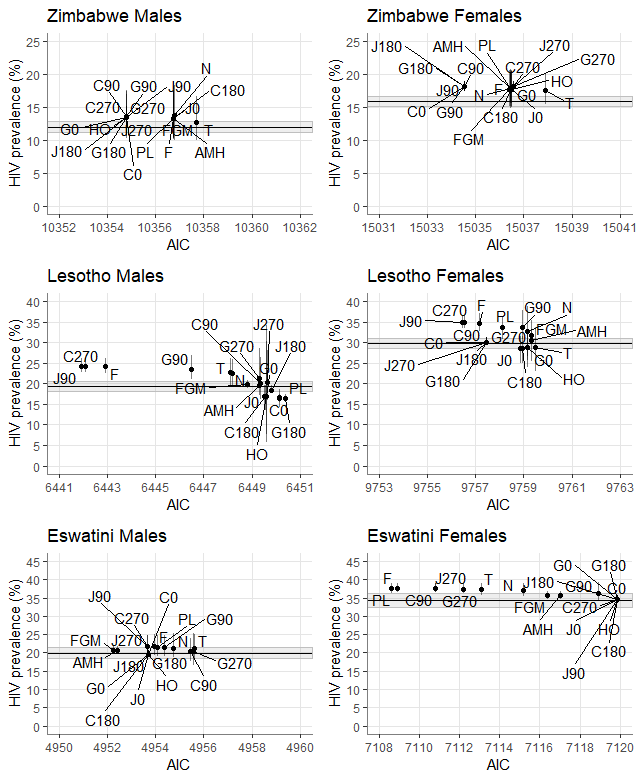


**Supplementary Figure 2b. Selection model results by copula**


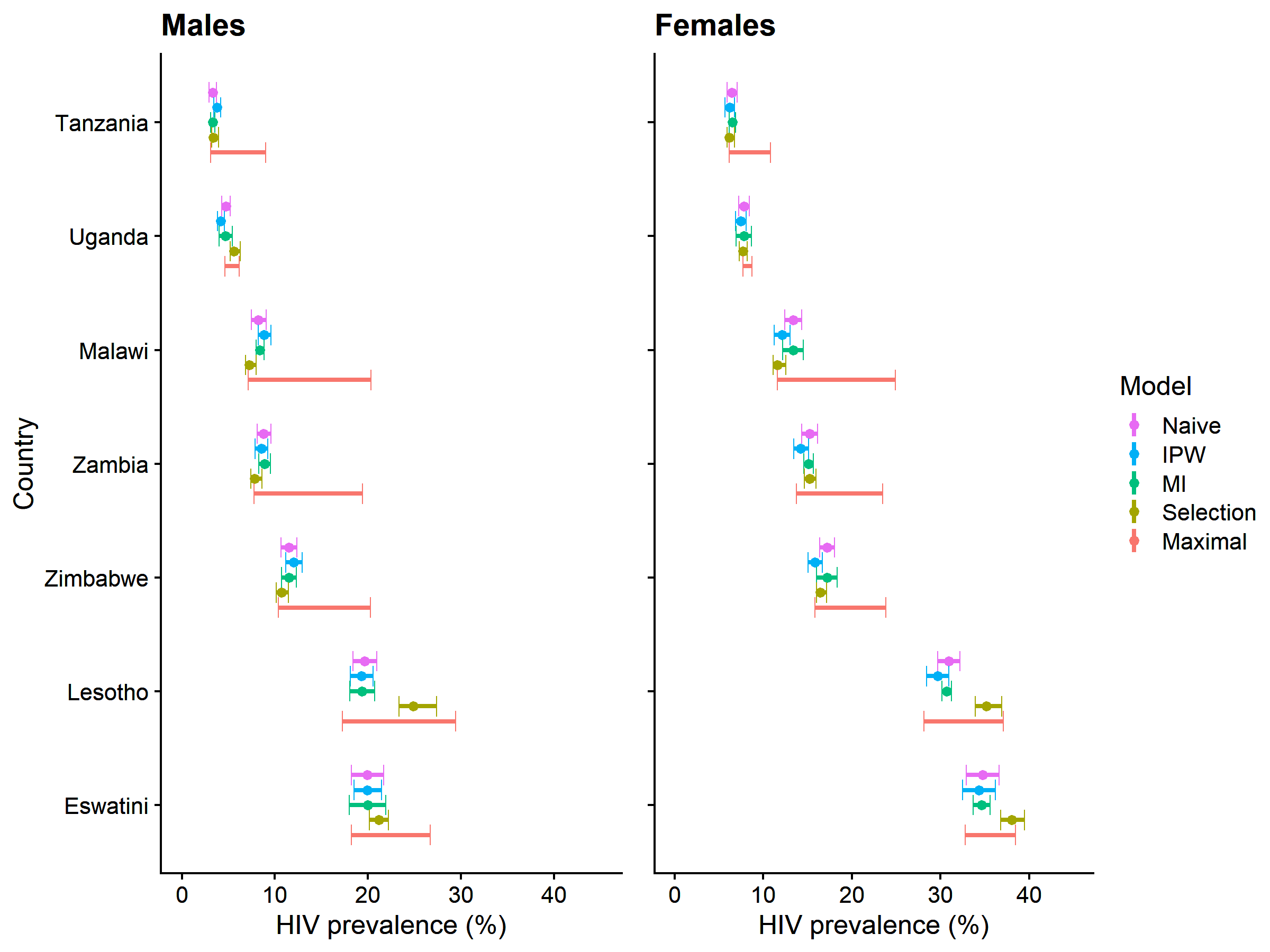


**Supplementary Figure 3. HIV prevalence estimates among adults aged 15-49 under different missingness assumptions using data from individual interview participants only.** Abbreviations: IPW: inverse probability weighting, MI: multiple imputation. Points and error bars represent HIV prevalence (%) and 95% CIs obtained from models under various missing data treatments using data from individual interview participants only, weighted to represent the national population. Maximal bounds are the theoretical minimum and maximum of possible HIV prevalence estimates in the sample assuming that all non-participants were HIV-negative or HIV-positive. Confidence intervals from other models may exceed the maximal bounds due to Taylor series variance approximation.

**Supplementary Table 4. HIV prevalence estimates among adults aged 15-49 under different missingness assumptions using data from individual interview participants only**

|  | **Country** |  |  |  |  |  |  |
| --- | --- | --- | --- | --- | --- | --- | --- |
| **Model** | **Tanzania** | **Uganda** | **Malawi** | **Zambia** | **Zimbabwe** | **Lesotho** | **Eswatini** |
|  | **Male** | | | | | | |
|  | N=10,971/11,531 | N=10,854/11,021 | N=6,306/7,283 | N=7,358/8,305 | N=7,241/8,020 | N=4,199/4,752 | N=3,655/3,988 |
| **Naive** | 3.3 (2.9, 3.8) | 4.7 (4.3, 5.2) | 8.2 (7.5, 9.1) | 8.8 (8.1, 9.6) | 11.5 (10.6, 12.4) | 19.6 (18.4, 21.0) | 19.9 (18.2, 21.7) |
| **IPW** | 3.8 (3.4, 4.2) | 4.2 (3.8, 4.6) | 8.9 (8.2, 9.6) | 8.5 (7.9, 9.2) | 12.0 (11.2, 12.9) | 19.3 (18.1, 20.6) | 19.9 (18.5, 21.5) |
| **MI** | 3.3 (3.1, 3.5) | 4.7 (4.0, 5.4) | 8.4 (8.0, 8.8) | 8.9 (8.3, 9.5) | 11.5 (10.7, 12.3) | 19.4 (18.0, 20.7) | 20.0 (18.0, 21.9) |
| **Selection** | 3.4 (3.2, 4.0) | 5.6 (5.2, 6.3) | 7.2 (6.8, 8.0) | 7.8 (7.4, 8.6) | 10.7 (10.1, 11.5) | 24.9 (23.3, 27.4) | 21.2 (20.1, 22.2) |
| **Maximal** | (3.1, 9.0) | (4.7, 6.2) | (7.2, 20.3) | (7.8, 19.4) | (10.3, 20.3) | (17.3, 29.4) | (18.2, 26.7) |
|  | **Female** | | | | | | |
|  | N=14,629/15,234 | N=14,716/14,860 | N=8,949/10,203 | N=10,010/11,052 | N=10,221/11,090 | N=5,990/6,536 | N=4,878/5,158 |
| **Naive** | 6.5 (6.0, 7.1) | 7.8 (7.2, 8.5) | 13.4 (12.4, 14.4) | 15.2 (14.4, 16.1) | 17.2 (16.4, 18.1) | 30.9 (29.7, 32.2) | 34.8 (32.9, 36.6) |
| **IPW** | 6.2 (5.7, 6.8) | 7.5 (6.9, 8.1) | 12.1 (11.3, 13.1) | 14.3 (13.5, 15.1) | 15.9 (15.1, 16.7) | 29.7 (28.5, 30.9) | 34.3 (32.5, 36.2) |
| **MI** | 6.5 (6.2, 6.9) | 7.8 (7.0, 8.7) | 13.4 (12.2, 14.6) | 15.1 (14.6, 15.7) | 17.2 (16.0, 18.4) | 30.7 (30.2, 31.3) | 34.6 (33.7, 35.6) |
| **Selection** | 6.2 (5.9, 6.8) | 7.7 (7.3, 8.2) | 11.6 (11.2, 12.5) | 15.2 (14.7, 16.0) | 16.4 (16.0, 17.1) | 35.2 (34.0, 36.9) | 38.0 (36.8, 39.5) |
| **Maximal** | (6.2, 10.8) | (7.8, 8.7) | (11.6, 24.9) | (13.8, 23.5) | (15.8, 23.9) | (28.2, 37.1) | (32.8, 38.4) |
|  | **Female-to-male prevalence ratio** | | | | | | |
| **Naive** | 1.97 | 1.66 | 1.63 | 1.73 | 1.50 | 1.58 | 1.75 |
| **IPW** | 1.63 | 1.79 | 1.36 | 1.68 | 1.33 | 1.54 | 1.72 |
| **MI** | 1.97 | 1.66 | 1.60 | 1.70 | 1.50 | 1.58 | 1.73 |
| **Selection** | 1.82 | 1.38 | 1.61 | 1.95 | 1.53 | 1.41 | 1.79 |
| Abbreviations: IPW: inverse probability weight, MI: multiple imputation. Note: N’s indicate the number of blood test participants over the number of eligible persons. The eligible population includes all de facto household members at the top and all individuals who participated in the individual interview at the bottom. | | | | | | | |


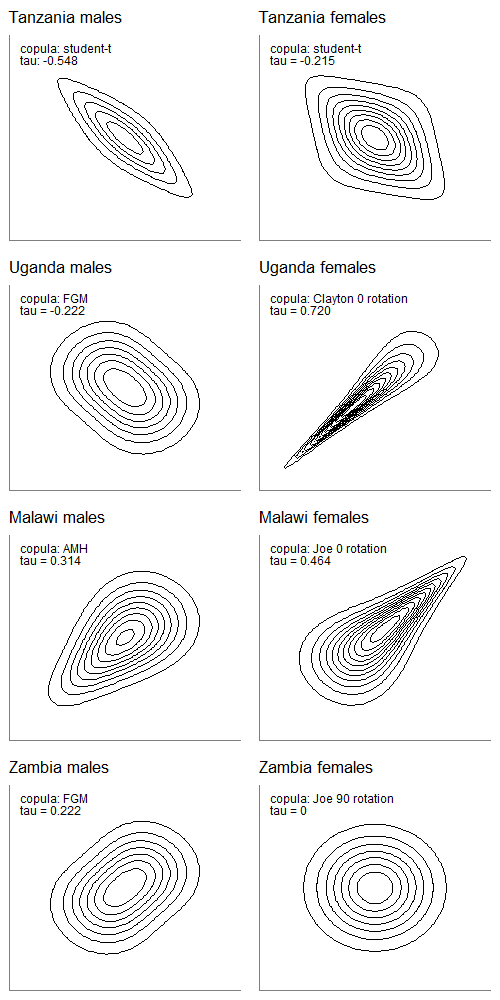


**Supplementary Figure 4a. Copula contour plots.** These plots illustrate the modeled relationship between the error terms in the selection (blood test participations; x-axis) and outcome (HIV status; y-axis) models. The preferred copula function is indicated at the top-left corner of each panel, alongside Kendall’s tau, a measure of the strength of the correlation. Negatively correlated distribution (pointing towards the top-left/bottom-right corners) indicates that those who are more likely to participate (further right on the x-axis) have lower likelihood of being HIV-positive (lower on the y-axis). Narrower distribution indicates stronger relationships between the selection and outcome.


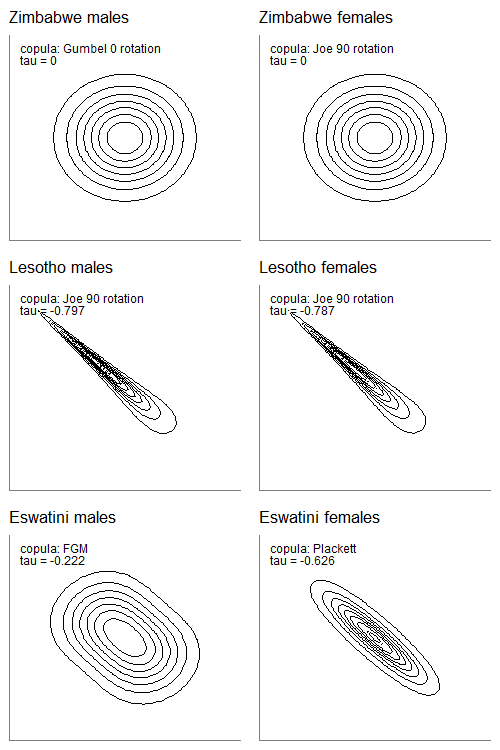


**Supplementary Figure 4b. Copula contour plots**
